# MIEF1/2 orchestrate mitochondrial dynamics through direct engagement with both the fission and fusion machineries

**DOI:** 10.1186/s12915-021-01161-7

**Published:** 2021-10-21

**Authors:** Rong Yu, Tong Liu, Shao-Bo Jin, Maria Ankarcrona, Urban Lendahl, Monica Nistér, Jian Zhao

**Affiliations:** 1 Department of Oncology-Pathology, Karolinska Institutet, BioClinicum, Visionsgatan 4, Karolinska University Hospital Solna, SE-171 64 Solna, Sweden; 2 Department of Cell and Molecular Biology, Karolinska Institutet, Biomedicum, Solnavägen 9, SE-171 77 Stockholm, Sweden; 3 Department of Neurobiology, Care Sciences and Society, Center for Alzheimer Research, Division of Neurogeriatrics, Karolinska Institutet, BioClinicum J9:20, Visionsgatan 4, SE-171 64 Solna, Sweden

**Keywords:** MIEF1/2, Mfn1/2, hFis1, Drp1, Mitochondrial dynamics, Mitochondrial fusion, Mitochondrial fission

## Abstract

**Background:**

Mitochondrial dynamics is the result of a dynamic balance between fusion and fission events, which are driven via a set of mitochondria-shaping proteins. These proteins are generally considered to be binary components of either the fission or fusion machinery, but potential crosstalk between the fission and fusion machineries remains less explored. In the present work, we analyzed the roles of mitochondrial elongation factors 1 and 2 (MIEF1/2), core components of the fission machinery in mammals.

**Results:**

We show that MIEFs (MIEF1/2), besides their action in the fission machinery, regulate mitochondrial fusion through direct interaction with the fusion proteins Mfn1 and Mfn2, suggesting that MIEFs participate in not only fission but also fusion. Elevated levels of MIEFs enhance mitochondrial fusion in an Mfn1/2- and OPA1-dependent but Drp1-independent manner. Moreover, mitochondrial localization and self-association of MIEFs are crucial for their fusion-promoting ability. In addition, we show that MIEF1/2 can competitively decrease the interaction of hFis1 with Mfn1 and Mfn2, alleviating hFis1-induced mitochondrial fragmentation and contributing to mitochondrial fusion.

**Conclusions:**

Our study suggests that MIEFs serve as a central hub that interacts with and regulates both the fission and fusion machineries, which uncovers a novel mechanism for balancing these opposing forces of mitochondrial dynamics in mammals.

**Supplementary Information:**

The online version contains supplementary material available at 10.1186/s12915-021-01161-7.

## Background

Mitochondria frequently change their morphology, size, and distribution via fission and fusion events to respond to altered metabolic demands or pathogenic assaults to the cell [1, 2]. Consequently, mitochondrial morphology is highly variable and takes on different shapes, such as small spheres, short tubules, and long tubular networks [3]. The dynamic changes in morphology, referred to as mitochondrial dynamics, are controlled by a set of mitochondria-shaping proteins through fission- and fusion-promoting programs, which counterbalance each other [1, 3–7].

Mitochondria-shaping proteins are generally classified as participating either in the mitochondrial fission or fusion machinery [7–11]. In mammalian cells, the mitochondrial fusion machinery contains three key components, including two mitochondrial outer membrane (MOM)-anchored dynamin-related GTPases, mitofusin 1 and 2 (Mfn1 and Mfn2), and one mitochondrial inner membrane (MIM)-anchored GTPase, optic atrophy 1 (OPA1). Mfn1 and Mfn2 are responsible for the fusion of the outer mitochondrial membranes [12–14], whereas OPA1 is responsible for the fusion of the inner membranes [15–17]. On the fission side, dynamin-related protein 1 (Drp1) together with Mff, MIEFs (MIEF1 and MIEF2), and Fis1 constitute the core components of the mammalian fission machinery [2, 18]. Drp1 plays a central role in the mitochondrial division [1, 3, 19], and it is a dynamin-related GTPase predominantly residing in the cytosol. Drp1 can be recruited from the cytosol to the mitochondrial surface, where it is assembled into higher-oligomeric ring complexes to wrap around the mitochondria, mediating mitochondrial division via its GTPase activity [18, 20]. However, unlike Mfns and OPA1, Drp1 does not contain any membrane-anchored domains, and recruitment of Drp1 to mitochondria therefore represents a crucial step in regulating Drp1-mediated mitochondrial division. The mode of Drp1 recruitment appears to differ between mammals and yeast. In mammalian cells, four MOM-anchored proteins, i.e., mitochondrial fission factor (Mff), mitochondrial elongation factor 1 and 2 (MIEF1 and MIEF2, also known as MiD51 and MiD49), and mitochondrial fission 1 (Fis1), are currently known to serve as receptors for recruiting Drp1 to mitochondria [1, 3]. In yeast, Fis1p is the major receptor for recruiting Dnm1p (the Drp1 ortholog in yeast) to mitochondria via one of the adaptor proteins Mdv1p and Caf4p [10, 21]. However, increasing evidence suggests that mammalian Fis1 is not essential for Drp1 recruitment [22], and overexpression or knockdown of Fis1 does not affect the distribution of Drp1 on mitochondria [23]. Instead, the three additional mammalian MOM-anchored proteins Mff, MIEF1, and MIEF2 play a major role in the recruitment of Drp1 to mitochondria [22, 24–26]. Knockdown of Mff reduces the association of Drp1 on mitochondria, resulting in mitochondrial elongation, whereas overexpression of Mff increases Drp1 recruitment to mitochondria, facilitating mitochondrial fragmentation [22].

How MIEFs act in mitochondrial dynamics is however more enigmatic. A number of reports support a role for MIEFs in mitochondrial fission. Notably, MIEFs interact with Drp1 in a manner independent of hFis1 and Mff [25, 26]. Increased expression of either MIEF1 or MIEF2 recruits Drp1 to the surface of mitochondria but blocks mitochondrial fission potentially via sequestering Drp1 into an inactive form on mitochondria, resulting in mitochondrial elongation [25–28]. Strikingly, knockdown of MIEFs however also causes mitochondrial elongation under most conditions [27–29]. A recent study further suggests that MIEFs can serve as molecular bridges between Drp1 and Mff, and in line with this notion depletion of MIEFs significantly reduces the association of Drp1 with Mff, thereby promoting mitochondrial elongation [29]. There are however also studies implying a role for MIEFs in promoting fusion. It was originally shown by us that elevated levels of MIEF1 reverse Mfn2 knockdown-induced mitochondrial fragmentation in 293T cells and facilitate mitochondrial fusion, causing mitochondrial elongation [25]. Along similar lines, Palmer et al. [30] reported that exogenous expression of MIEF1 or MIEF2 in mouse embryonic fibroblasts (MEFs) reverses Mfn1 or Mfn2 deficiency-induced mitochondrial fragmentation, resulting in mitochondrial elongation. The mitochondrial elongation caused by high MIEF1/2 expression was presumed to be due to sequestration and inactivation of Drp1 on mitochondria, blocking fission and leading to unopposed mitochondrial fusion [30]. Thus, whether MIEFs directly participate in the regulation of mitochondrial fusion still remains controversial.

In this report, we analyze the molecular basis for the action of MIEFs in mitochondrial fusion. We find that MIEFs interact with the pro-fusion proteins Mfn1 and Mfn2 and that elevated levels of MIEF1 or MIEF2 facilitate mitochondrial fusion irrespective of whether Drp1 is present or not. Furthermore, we show that MIEFs can affect hFis1’s interaction with Mfn1 and Mfn2 in the fusion machinery, thereby abrogating the inhibitory effect of hFis1 on fusion and reversing hFis1-induced mitochondrial fragmentation, resulting in mitochondrial elongation. Finally, mitochondrial localization and self-association of MIEFs are indispensable for the fusion-promoting ability of MIEFs. In summary, our data show that MIEFs interact with the major components in both the fission and fusion machineries and serve as a hub for regulating the balance between fusion and fission in mammalian cells.

## Results

### MIEFs are part of both the mitochondrial fission and fusion machineries

**Fig. 1:**
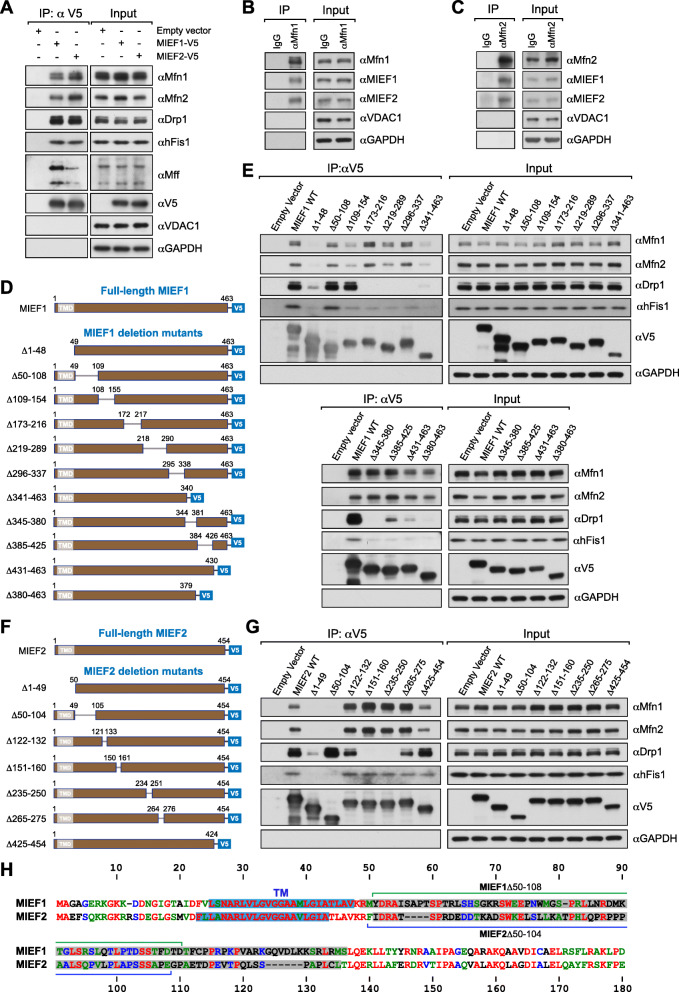
Identification and characterization of binding regions in MIEFs required for Mfn1, Mfn2, hFis1, and Drp1. **a** Exogenous MIEF1-V5 and MIEF2-V5 interact with both pro-fission (Drp1, hFis1, Mff) and pro-fusion (Mfn1, Mfn2) proteins. 293T cells were transfected with empty vector (as control), MIEF1-V5, or MIEF2-V5 and were in vivo crosslinked with 1% formaldehyde (FA). Cell lysates were used for co-IP with anti-V5 beads, followed by Western blotting with indicated antibodies. **b**, **c** Mfn1 and Mfn2 interact with MIEF1/2 at endogenous levels. 293T cells were in vivo crosslinked with 1% FA. Cell lysates were subjected to co-IP with Protein G beads pre-bound to mouse/rabbit normal IgG (negative control), anti-Mfn1 (**b**), or Mfn2 (**c**) antibodies, followed by immunoblotting with indicated antibodies. **d** Summary of MIEF1-mutated constructs used in the co-IP experiments. **e** Interaction of different MIEF1 mutants with Mfn1, Mfn2, Drp1, and hFis1. 293T cells were transfected with full-length MIEF1 and deletion mutants as indicated, and in vivo crosslinked with 1% FA. Cell lysates were used for co-IP with anti-V5 beads, followed by Western blotting with indicated antibodies. **f** Summary of MIEF2-mutated constructs used in the co-IP experiments. **g** Interaction of different MIEF2 mutants with Mfn1, Mfn2, Drp1, and hFis1. 293T cells were transfected with the full-length and the truncated MIEF2 mutants as indicated, and in vivo crosslinked with 1% FA. Cell lysates were used for co-IP with anti-V5 beads, followed by Western blotting with indicated antibodies. **h** Protein sequence alignments around the transmembrane domains (TM, in blue color) and the disordered regions (DR, gray color) comparing human MIEF1 (NP_061881) and MIEF2 (NP_631901). The amino acid sequence alignments of human MIEF1 and MIEF2 were generated by the CLUSTALW program (https://npsa-prabi.ibcp.fr). The regions that were deleted in MIEF1^Δ50-108^ and MIEF2^Δ50-104^ are indicated in the alignment. Red: identical; Green: strongly similar; Blue: weakly similar; Black: different amino acids

In order to explore the potential relationship between MIEFs and the mitochondrial fusion apparatus, we performed co-immunoprecipitation (co-IP) followed by immunoblotting analysis in 293T cells transiently expressing MIEF1-V5 or MIEF2-V5 after in vivo chemical crosslinking as previously described [25, 31]. In keeping with the results above, apart from the interaction with the pro-fission proteins Drp1, hFis1, and Mff as previously reported [25, 26, 29], we found that the key pro-fusion GTPases Mfn1 and Mfn2 were both co-precipitated efficiently with exogenously expressing MIEF1-V5 and MIEF2-V5 (Fig. 1a). Similarly, at endogenous levels, MIEF1 and MIEF2 could be co-immunoprecipitated with beads pre-incubated with antibodies against Mfn1 and Mfn2 following in vivo chemical crosslinking (Fig. 1b, c). In line with these results, MIEFs still robustly interacted with Mfn1 and Mfn2 in the absence of chemical crosslinking (Additional file 1, Figure S1a, b). Additionally, we used VDAC1 (voltage-dependent anion channel 1), a mitochondrial outer membrane protein as a negative control in the co-IP experiments, and no interaction between MIEF1/2 and VDAC1 was detected (Fig. 1a–c; see also Additional file 1, Figure S1a, b). Altogether, the data suggest that MIEFs are components of not only the fission but also the fusion machinery.

### Characterization of binding regions in MIEF1 and MIEF2 responsible for Mfn1, Mfn2, and Drp1 interactions

In order to further characterize protein-binding regions in MIEFs required for their interaction with Mfn1, Mfn2, hFis1, and Drp1, we constructed a series of V5-tagged deletion mutants of MIEF1 (Fig. 1d; see also Additional file 1, Figure S2) and MIEF2 (Fig. 1f; see also Additional file 1, Figure S3) and performed co-IP experiments following chemical crosslinking. We first determined whether mitochondrial localization of MIEFs was essential for interaction with Mfn1, Mfn2, hFis1, and Drp1. Co-IP followed by immunoblotting showed that the cytosolic mutant MIEF1^Δ1-48^, which lacks the N-terminal first 48 residues including the TM (transmembrane) domain, severely impaired the interaction with Mfn1 and Mfn2, as well as the association with hFis1 and Drp1 (Fig. 1d, e; summarized in Table 1). Similar to MIEF1, the deletion of the first 49 amino acid residues including the TM domain of MIEF2 at the N-terminus (i.e., the cytosolic mutant MIEF2^Δ1-49^) caused an almost complete loss of the ability to interact with Mfn1, Mfn2, and hFis1 and also resulted in a severe decrease in the ability to bind Drp1 (Fig. 1f, g; summarized in Table 1). These results suggest the mitochondrial localization of MIEFs is important for binding to Mfn1, Mfn2, and Drp1.

**Table 1.**
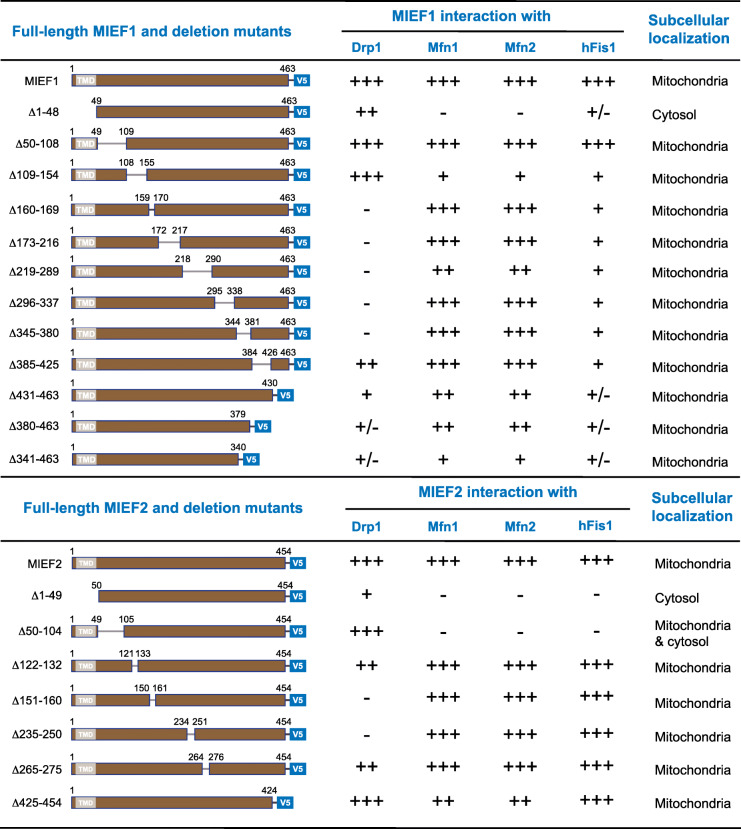
Summary of binding regions in MIEF1/2 required for interactions with Drp1, Mfn1, Mfn2 and hFis1.

Summary of potential protein binding regions in MIEFs required for Drp1, Mfn1, Mfn2 and hFis1 interactions identified by deletion mutants as presented in Figure 1. For the MIEF1Δ160-169 co-IP, see Fig 4B. Notice: -, +/-, +, ++ and +++ stand for the interaction from weakest to strongest as identified by co-IP followed by Western blotting.

We next evaluated whether the disordered regions (DR) in MIEF1 and MIEF2 are required for interaction with Mfn1, Mfn2, and Drp1. Our results showed that an internal deletion of the residues from 50 to 108 (MIEF1^Δ50-108^), located in the DR (residues 48–134) close to the TM domain of MIEF1, as described in structural studies [32, 33], did not affect its association with Mfn1, Mfn2, hFis1, and Drp1, as compared to wild-type MIEF1 (Fig. 1d, e; summarized in Table 1). However, unlike the situation in MIEF1, the DR (residues 51–125) in MIEF2 [34] was more important for its interaction with Mfn1/2. Deletion of this region in MIEF2 (such as MIEF2^Δ50-104^) greatly diminished its association with Mfn1, Mfn2, and hFis1 but did not affect its interaction with Drp1 (Fig. 1f, g; summarized in Table 1). These findings suggest that the DR of MIEF2 is responsible for its interaction with Mfn1/2 and hFis1 and is therefore functionally distinct from the DR of MIEF1. Consistent with this, amino acid sequences of the DR are quite diverged between MIEF1 and MIEF2 (Fig. 1h), but the DRs in MIEF1 as well as in MIEF2 were found to be evolutionarily highly conserved in different species (see Additional file 1, Figures. S2 and S3), indicating potentially important and diverged biological functions of the DRs in MIEF1 and MIEF2.

We next tested whether other regions in MIEF1 and MIEF2 are required for interaction with Mfn1 and Mfn2. Co-IP assays revealed that deletion of the last 32 residues at the C-terminus (the mutant MIEF1^Δ431-463^) resulted in a severe decrease in the ability of MIEF1 to bind Mfn1/2, while further deletion of the C-terminal regions (such as MIEF1^Δ380-463^ and MIEF1^Δ341-463^) did not further impair the interaction with Mfn1/2 (Fig. 1d, e; summarized in Table 1). Additionally, the two internal deletion mutants MIEF1^Δ109-154^ and MIEF1^Δ219-289^ also exhibited decreased interaction with Mfn1 and Mfn2 (Fig. 1d, e; summarized in Table 1). However, unlike in MIEF1, all the MIEF2 deletion mutants analyzed, except MIEF2^Δ1-49^ and MIEF2^Δ50-104^, retained the ability to bind Mfn1 and Mfn2 (Fig. 1f, g; summarized in Table 1).

Subsequently, we mapped the regions in MIEF1 necessary for interaction with hFis1. We found that all the deletion mutants analyzed, except MIEF1^Δ50-108^ located in the DR of MIEF1, impaired but did not completely abolish MIEF1’s interaction with hFis1 (Fig. 1d, e; summarized in Table 1). However, unlike MIEF1, all the MIEF2 deletion mutants analyzed, except MIEF2^Δ1-49^ and MIEF2^Δ50-104^, retained the ability to bind Mfn1, Mfn2, OPA1, and hFis1 (Fig. 1f, g; summarized in Table 1).

We also used the set of MIEF1 and MIEF2 deletion mutants to more precisely define the binding region between MIEF1/2 and Drp1. It has previously been shown that deletion of residues 160–169 in MIEF1 (MIEF1^Δ160-169^) led to a complete loss of the ability to bind Drp1 [25]. Likewise, co-IP assays revealed that the internal deletion mutants MIEF1^Δ173-216^, MIEF1^Δ219-289^, MIEF1^Δ296-337^, and MIEF1^Δ345-380^ exhibited a complete loss of interaction between MIEF1 and Drp1 (Fig. 1d, e; summarized in Table 1), indicating that the middle region of MIEF1 from residues 160 to 380 is required for the binding to Drp1. In addition, the C-terminal region of MIEF1 might also be involved in the interaction with Drp1, because the mutant MIEF1^Δ431-463^, lacking the last 32 residues at the C-terminus, showed a severely impaired interaction with Drp1, and further deletion of the C-terminal region (such as MIEF1^Δ380-463^ and MIEF1^Δ341-463^) gave rise to a further reduction in binding to Drp1 (Fig. 1d, e; summarized in Table 1), in line with a recent structural study reporting that the C-terminal region of MIEF1 (420–451aa) is involved in Drp1 binding [35].

The middle region of MIEF2, probably including residues 151 to 250, is potentially required for binding to Drp1, because the internal deletion mutants MIEF2^Δ151-160^ and MIEF2^Δ235-250^ displayed a complete loss of MIEF2’s binding to Drp1 (Fig. 1f, g; summarized in Table 1). However, one important difference between MIEF1 and MIEF2 is that the C-terminal deletion of MIEF2 (e.g., mutant MIEF2^Δ425-454^, lacking the last 29 residues at the C-terminus) retained its interaction with Drp1 (Fig. 1f, g), while the C-terminal deletion of MIEF1 (e.g., mutant MIEF1^Δ431-463^) substantially reduced the association with Drp1 (Fig. 1d, e).

Finally, we assessed the potential cytotoxic effect (apoptosis) of the MIEF mutants. We showed that overexpression of WT MIEF1 or MIEF2 as well as their deletion mutants did not affect the levels of cleaved PARP (Additional file 1, Figure S4a, b).

### MIEFs partially reverse mitochondrial fragmentation caused by knockdown of Mfn1 or Mfn2

**Fig. 2:**
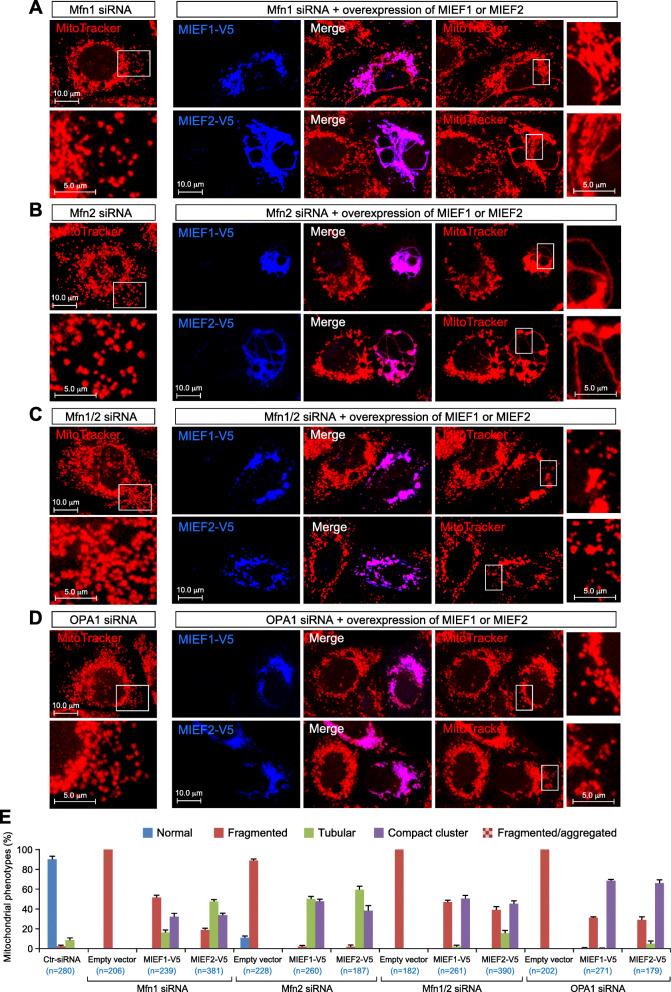
Expression of MIEF1-V5 or MIEF2-V5 partially reverses mitochondrial fragmentation induced by knockdown of Mfn1, Mfn2, or OPA1. **a–d** Confocal images of mitochondrial morphology in 293T cells treated with Mfn1 (**a**), Mfn2 (**b**), Mfn1 plus Mfn2 (**c**), or OPA1 (**d**) siRNA, followed by transfection with empty vector, MIEF1-V5, or MIEF2-V5 as indicated. The cells were stained with MitoTracker (red) followed by immunostaining with anti-V5 (blue) antibody. **e** Percentages (mean ± SEM) of cells with indicated mitochondrial morphologies in 293T cells as shown in (**a**–**d**) in three independent experiments for each condition (*n* represents the total number of cells analyzed for each condition)

The robust interaction with the pro-fusion GTPases Mfn1/2 in the fusion machinery suggests that MIEFs may play roles in regulating mitochondrial fusion. To address this issue, we first asked whether elevated levels of MIEFs could reverse mitochondrial fragmentation caused by the knockdown of key components in the fusion machinery. We tested this by overexpressing MIEF1 and MIEF2 in combination with siRNA-mediated knockdown of Mfn1, Mfn2, and OPA1 alone or in combination. Western blotting showed that protein levels of endogenous Mfn1, Mfn2, and OPA1 were reduced by over 90% after treatment with the specific siRNAs (Additional file 1, Figure S5a, b). In normal 293T cells (treated with control siRNA), mitochondria appeared as a mixed reticulum of tubular and round forms (Additional file 1, Figure S5c, upper left panel). In contrast, knockdown of either Mfn1, Mfn2, or OPA1 alone, or simultaneous knockdown of Mfn1 and Mfn2 by siRNA, resulted in extensive mitochondrial fragmentation (Fig. 2a–d, left panels, summarized in Fig. 2e; see also Additional file 1, Figure S5c), confirming previous reports [36–38]. Increased expression of MIEF1 or MIEF2 efficiently reversed mitochondrial fragmentation induced by knockdown of either Mfn1 (Fig. 2a, right panels) or Mfn2 (Fig. 2b, right panels), and significantly increased the number of cells with a tubular mitochondrial phenotype compared to knockdown of Mfn1 and Mfn2 alone (summarized in Fig. 2e; see also Additional file 1, Figure S5c). However, exogenous expression of MIEF1 or MIEF2 did not reverse mitochondrial fragmentation induced by simultaneous knockdown of endogenous Mfn1 and Mfn2 (Fig. 2c, right panels, summarized in Fig. 2e; see also Additional file 1, Figure S5c) or by knockdown of OPA1 (Fig. 2d, right panels, summarized in Fig. 2e), which is in line with previous reports [25, 30]. Collectively, the data show that elevated levels of MIEF1 or MIEF2 can partially compensate for knockdown of Mfn1 or Mfn2 alone, but not for Mfn1/2 double knockdown or OPA1 knockdown, with regard to mitochondrial fragmentation.

### Exogenous expression of MIEF1 or MIEF2 enhances mitochondrial fusion independently of their inhibitory effect on Drp1

**Fig. 3:**
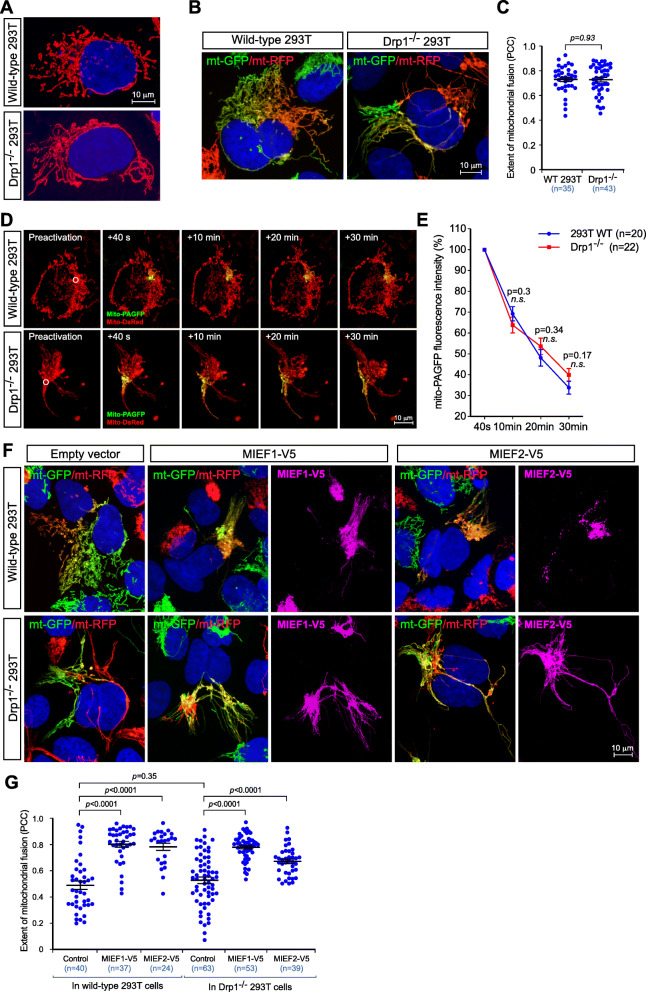
MIEFs promote mitochondrial fusion independent of Drp1. **a** Confocal images of mitochondrial morphology in wild-type (WT) and Drp1^−/−^ 293T cells. **b**, **c** Confocal images of mitochondrial fusion in WT and Drp1^−/−^ 293T polykaryons from the PEG-mediated cell fusion assay. Cells stably expressing mitoRFP (red) or mitoGFP (green) were co-cultured and subjected to PEG-mediated cell fusion. Mitochondrial fusion is indicated by co-localization of mitoRFP and mitoGFP (yellow mitochondria) (**b**). The extent of mitochondrial fusion in individual hybrid cells was analyzed by Pearson’s correlation coefficient (PCC) in three independent experiments for each condition and data are summarized in (**c**). **d**, **e** The mito-PAGFP-based mitochondrial fusion assay in WT and Drp1^−/−^ 293T cells. Cells co-transfected with mito-PAGFP (0.5 μg) and mito-DsRed (0.2 μg) were photoactivated in the ROI (white circle, 3 μm diameter) in preactivation images of mitochondria (red). After photoactivation, mito-PAGFP fluorescence (green) intensity and mitochondrial marker (mito-DsRed) were collected at indicated time points (**d**). Mitochondrial fusion was quantified by analyzing changes in fluorescence intensity of photoactivated mito-PAGFP in ROIs at 40 s and 10, 20, and 30 min. The dilution rates (percentage) of the GFP fluorescence intensity at different time points were normalized by the fluorescence intensity at 40 s after photoactivation (**e**). (*n* represents the total number of cells analyzed for each condition). *n.s.*, not significant. **f**, **g** Confocal images of mitochondrial fusion from the PEG-based fusion assay in WT and Drp1^−/−^ 293T polykaryons transfected with empty vector, MIEF1-V5, or MIEF2-V5. Cells with stable expression of mitoRFP or mitoGFP were co-cultured and transfected with empty vector (control), MIEF1-V5, or MIEF2-V5 as indicated and then subjected to PEG-mediated cell fusion. Mitochondrial fusion is indicated by co-localization of mitoRFP (red) and mitoGFP (green) (yellow mitochondria) (**f**). The extent of mitochondrial fusion in individual hybrid cells was analyzed via the Pearson’s correlation coefficient (PCC) in three independent experiments for each condition and data summarized in (**g**). Data are expressed as means ± SEM, *n* represents the number of cells analyzed (**c**, **e**, and **g**)

In order to functionally dissect the role of MIEFs in mitochondrial fusion, we next asked whether their Drp1-inhibiting function was required. To this end, we used wild-type (WT) and Drp1-deficient (Drp1^−/−^) 293T cells, in which we stably expressed either mitoGFP (mitochondria-targeted GFP) (green) or mitoRFP (mitochondria-targeted RFP) (red) as described previously [31] and performed a series of polyethylene glycol (PEG)-mediated cell fusion experiments. In WT 293T cells, mitochondria appeared as a mixed reticulum with tubular and round forms (Fig. 3a, upper panel), whereas mitochondria in Drp1^−/−^ 293T cells displayed the expected super-elongated tubular network (Fig. 3a, lower panel). As the increasing expression of MIEF1 or MIEF2 is known to result in the elongated mitochondrial phenotype likely by inhibition of Drp1 function [25–27, 30], we first evaluated whether the presence or absence of endogenous Drp1 affected MIEF1/2-induced mitochondrial fusion. To test this, mitoGFP- and mitoRFP-labeled WT 293T cells, as well as mitoGFP- and mitoRFP-labeled Drp1^−/−^ 293T cells were mixed and cultured overnight, and the PEG-induced cell fusion assay was performed as previously described [25, 31]. Mitochondrial fusion was revealed by measuring the extent of mixing of green and red fluorescence (yellow) in the polykaryons by Pearson’s correlation coefficient (PCC). The results show that the extent of mitochondrial fusion was similar between WT 293T and Drp1^−/−^ 293T cells (Fig. 3b, c), indicating that loss of Drp1 results in mitochondrial elongation, but does not affect mitochondrial fusion.

To further corroborate the results described above, we performed a mitochondrial matrix-targeted photoactivatable green fluorescent protein (mito-PAGFP)-based mitochondrial fusion assay [39], in which mito-PAGFP was used to monitor and quantify the alteration of mitochondrial fusion in WT 293T cells and Drp1^−/−^ 293T cells at different time points. After photoactivation of a small region of interest (ROI), diffusion and dilution of the photoactivated GFP fluorescence within the mitochondrial network were followed in a time course experiment. The decrease of GFP fluorescence intensity within the photoactivated ROI at different time points was used for measuring mitochondrial fusion rates [40]. The mito-PAGFP fusion assay confirmed that the rate of mitochondrial fusion was not affected by the presence or absence of Drp1 (Fig. 3d, e).

**Fig. 4:**
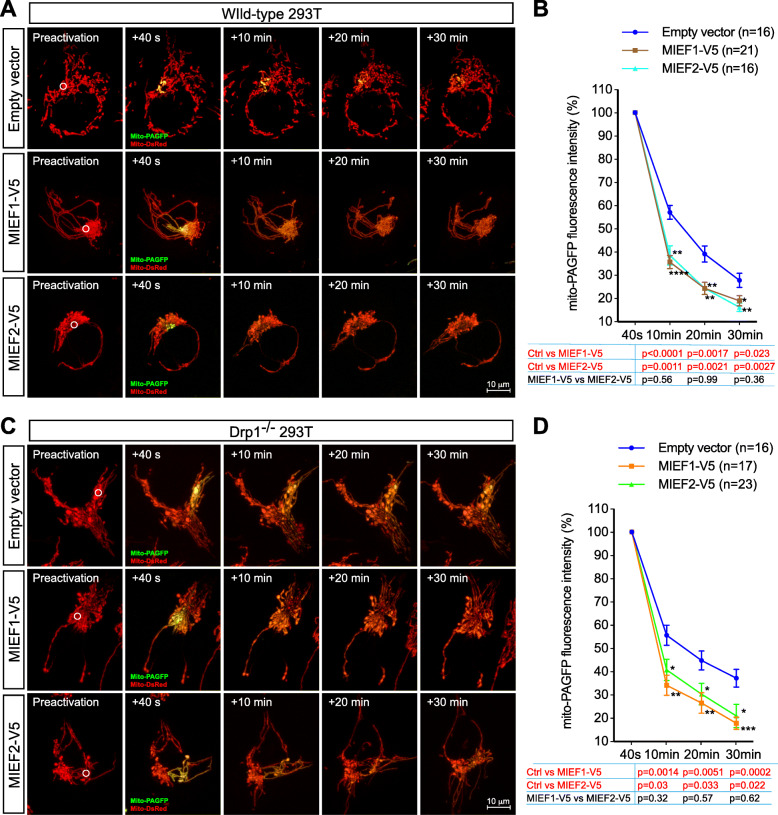
MIEF-mediated mitochondrial fusion is validated by the mito-PAGFP-based mitochondrial fusion assay in WT and Drp1^−/−^ 293T cells. **a**–**d** The mito-PAGFP-based mitochondrial fusion assay in WT 293T (**a**, **b**) and Drp1^−/−^ 293T (**c**, **d**) cells. Cells co-transfected with mito-PAGFP (0.5 μg), mito-DsRed (0.2 μg), and either empty vector (0.5 μg), MIEF1 (0.5 μg), or MIEF2 (0.5 μg) were photoactivated in the ROI (white circle, 3 μm diameter) in preactivation images of mitochondria (red). After photoactivation, mito-PAGFP fluorescence (green) intensity and mitochondrial marker (mito-DsRed) were collected at indicated time points (**a**, **c**). Mitochondrial fusion was quantified by analyzing changes in fluorescence intensity of photoactivated mito-PAGFP in ROIs at 40 s and 10, 20, and 30 min. The dilution rates (percentage) of the GFP fluorescence intensity at different time points were normalized by the fluorescence intensity at 40 s after photoactivation (**b**, **d**) (*n* represents the number of cells analyzed). ****P < 0.0001; ***P < 0.001; **P < 0.01; *P < 0.05

We next assessed the effect of MIEF1/2 on mitochondrial fusion. Both the PEG-mediated cell fusion assay (Fig. 3f, g) and the mito-PAGFP-based fusion assay (Fig. 4a–d) revealed that the increasing expression of MIEF1 or MIEF2 significantly enhanced the extent of mitochondrial fusion in WT 293T as well as in Drp1^−/−^ 293T cells compared to controls (transfected with empty vector). These data indicate that MIEF1/2 can efficiently facilitate mitochondrial fusion regardless of whether Drp1 is present in the cells or not.

**Fig. 5:**
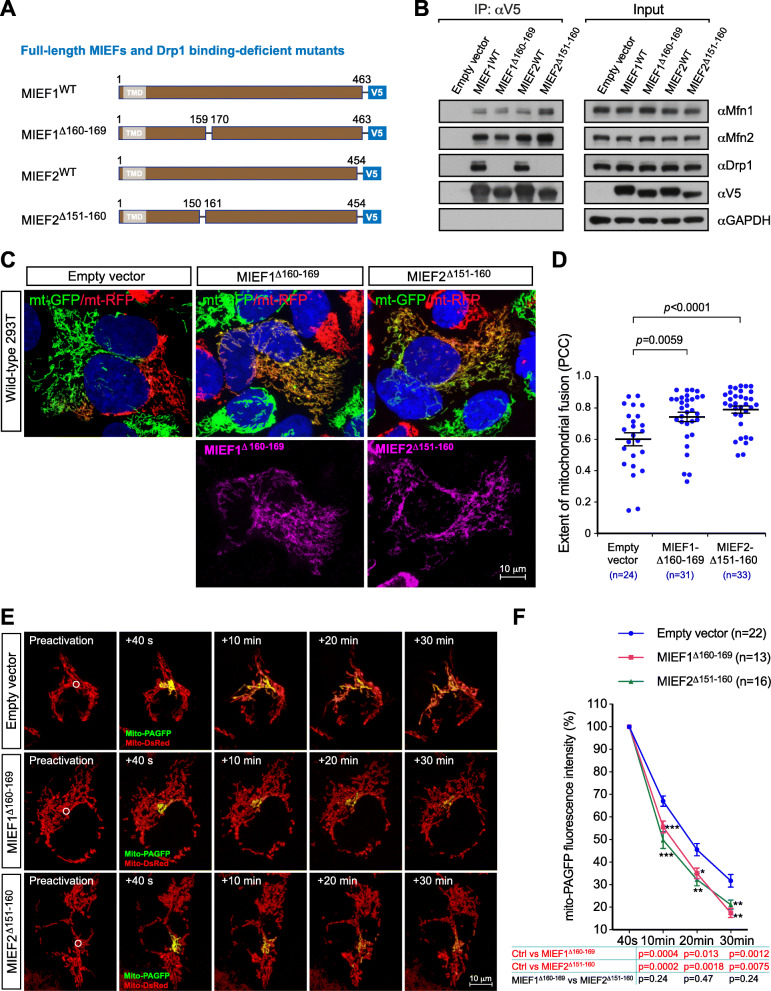
Drp1 binding-deficient mutants of MIEFs interact with pro-fusion proteins and promote mitochondrial fusion. **a** Schematic representation of full-length MIEF1 and MIEF2, as well as their Drp1 binding-deficient mutants fused to V5-tag at the C terminus. **b** Drp1 binding-deficient mutants of MIEFs still interact with Mfn1 and Mfn2. 293T cells were transfected with empty vector, MIEF1-V5, MIEF1^Δ160-169^-V5, MIEF2-V5, or MIEF2^Δ151-160^-V5. Cell lysates were used for co-IP with anti-V5 beads, followed by Western blotting with indicated antibodies. **c**, **d** Representative confocal images of mitochondrial fusion from the PEG-based fusion assay in WT 293T polykaryons transfected with empty vector, MIEF1^Δ160-169^ or MIEF2^Δ151-160^. Cells with stable expression of mitoRFP or mitoGFP were co-cultured, and transfected with empty vector (control), MIEF1^Δ160-169^-V5 or MIEF2^Δ151-160^-V5 as indicated, then subjected to the PEG cell fusion assay. The mitochondrial fusion is indicated by co-localization of mitoRFP and mitoGFP (i.e., yellow mitochondria) (**c**). The extent of mitochondrial fusion in individual hybrid cells was analyzed via the Pearson’s correlation coefficient (PCC, mean ± SEM) in three independent experiments for each condition and data are summarized in (**d**) (*n* represents the total number of cells analyzed for each condition). **e**, **f** The mito-PAGFP-based mitochondrial fusion assay confirmed that Drp1 binding-deficient mutants MIEF1^Δ160-169^ or MIEF2^Δ151-160^ still enhanced mitochondrial fusion. *n* represents the number of cells analyzed. ***P < 0.001; **P < 0.01; *P < 0.05 (**f**)

To further corroborate the notion that Drp1 is not required for MIEF-induced mitochondrial fusion, we used two previously generated Drp1 binding-deficient deletion mutants, MIEF1^Δ160-169^ and MIEF2^Δ151-160^ (Fig. 5a) [25, 29], and evaluated their effects on mitochondrial fusion using the PEG-mediated cell fusion assay. As shown in Fig. 5b, co-IP experiments demonstrated that both MIEF mutants had completely lost the ability to bind Drp1, in line with our previous reports [25, 29], but still retained the capacity to interact with the pro-fusion GTPases Mfn1 and Mfn2. 293T cell lines stably expressing mitoGFP (green) or mitoRFP (red) were mixed and cultured overnight, and then transfected with empty vector (as control) or plasmids with either V5-tagged MIEF1^Δ160-169^ or V5-tagged MIEF2^Δ151-160^, subjected to the PEG-mediated cell fusion assay. As shown in Fig. 5c, d, both Drp1 binding-deficient mutants could still facilitate mitochondrial fusion in the PEG-mediated cell fusion assay. To further validate this result, we performed the mito-PAGFP-mediated mitochondrial fusion assay, which confirmed that the MIEF1^Δ160-169^ and MIEF2^Δ151-160^ mutants enhanced mitochondrial fusion (Fig. 5e, f). Together, these data underscore that the interaction between MIEFs and Drp1 is not required for MIEF1/2-induced mitochondrial fusion. In addition, we showed that ablation of MIEF1 or MIEF2 in 293T cells by CRISPR/Cas9-mediated genome editing [29] did not significantly affect mitochondrial fusion compared to WT 293T cells. However, re-introduction of MIEF1-V5 or MIEF2-V5 enhanced mitochondrial fusion, as evaluated by the mito-PAGFP-mediated mitochondrial fusion assay (Additional file 1, Figure S6).

### Mitochondrial localization and dimerization/oligomerization of MIEFs are required for their fusion-promoting ability

**Fig. 6:**
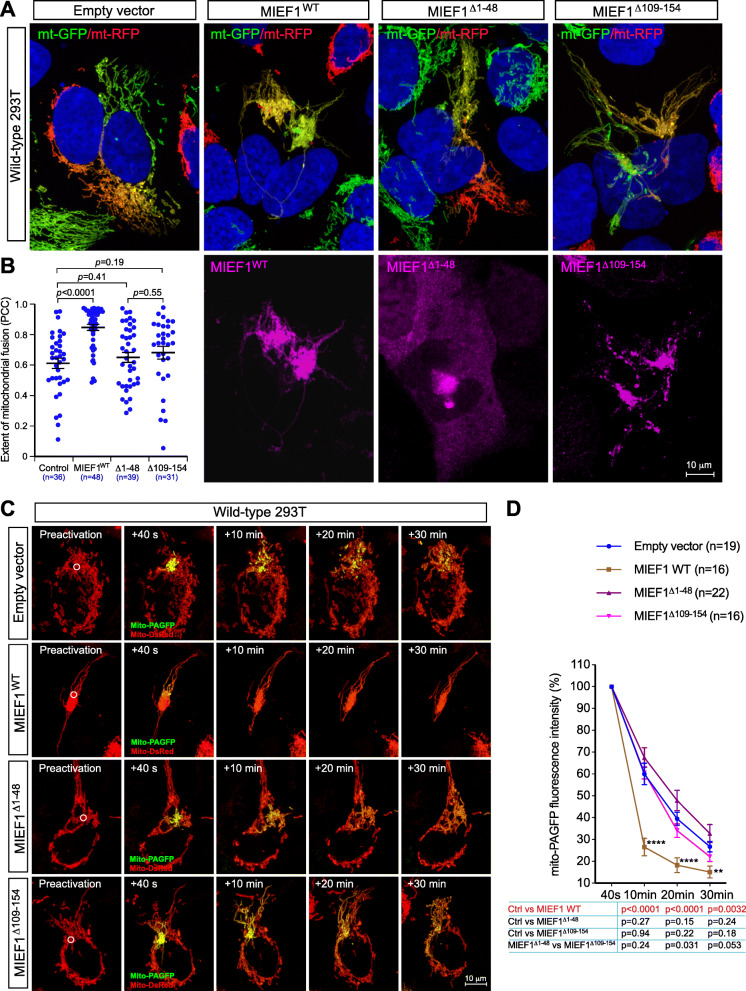
MIEF self-association is required for MIEF-induced mitochondrial fusion. **a**, **b** Confocal images of mitochondrial fusion from the PEG-based fusion assay in WT 293T polykaryons transfected with empty vector, MIEF1-V5, MIEF1^Δ1-48^-V5, or MIEF1^Δ109-154^-V5. Cells with stable expression of mitoRFP or mitoGFP were co-cultured, transfected with empty vector, MIEF1-V5 (WT), MIEF1^Δ1-48^-V5, or MIEF1^Δ109-154^-V5 as indicated, and then subjected to the PEG cell fusion assay. Mitochondrial fusion is indicated by co-localization of mitoRFP (red) and mitoGFP (green) (i.e., yellow mitochondria) (**a**, upper panels). As indicated by V5-tag staining, the MIEF1^Δ1-48^ mutant localizes in the cytosol, whereas MIEF1^Δ109-154^ is on mitochondria (**a**, lower panels). The extent of mitochondrial fusion in individual hybrid cells was analyzed via the Pearson’s correlation coefficient (PCC, mean ± SEM) in three independent experiments for each condition and data summarized in (**b**) (*n* represents the total number of cells analyzed for each condition). **c**, **d** The mito-PAGFP-based mitochondrial fusion assay confirmed that the cytosolic mutant MIEF1^Δ1-48^ and the self-association-deficient mutant MIEF1^Δ109-154^ lost the ability to enhance mitochondrial fusion. *n* represents the number of cells analyzed. ****P < 0.0001; **P < 0.01 (**d**)

**Fig. 7:**
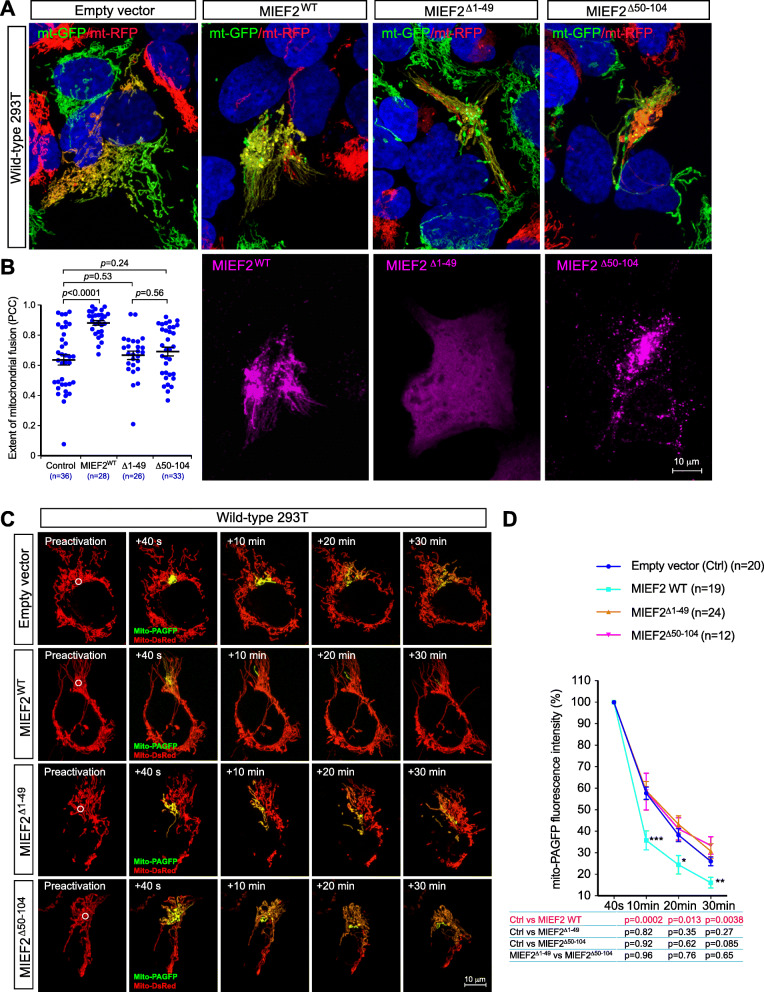
MIEF self-association is required for MIEF-induced mitochondrial fusion. **a**, **b** Confocal images of mitochondrial fusion from the PEG-based fusion assay in WT 293T polykaryons transfected with empty vector, MIEF2-V5 (WT), MIEF2^Δ1-49^-V5, or MIEF2^Δ50-104^-V5. Cells with stable expression of mitoRFP or mitoGFP were co-cultured, transfected with empty vector, MIEF2-V5, MIEF2^Δ1-49^-V5, or MIEF2^Δ50-104^-V5 as indicated and then subjected to the PEG cell fusion assay. Mitochondrial fusion is indicated by co-localization of mitoRFP and mitoGFP (i.e., yellow mitochondria) (**a**, upper panels). As indicated by V5-tag staining, the MIEF2^Δ1-49^ mutant localizes in the cytosol, whereas MIEF2^Δ50-104^ is mainly on mitochondria (**a**, lower panels). The extent of mitochondrial fusion in individual hybrid cells was analyzed via the Pearson’s correlation coefficient (PCC, mean ± SEM) in three independent experiments for each condition and data are summarized in (**b**) (*n* represents the total number of cells analyzed for each condition). **c**, **d** The mito-PAGFP-based mitochondrial fusion assay confirmed that the cytosolic mutant MIEF2^Δ1-49^ and the self-association-deficient mutant MIEF1^Δ50-104^ lost the ability to enhance mitochondrial fusion. *n* represents the number of cells analyzed. ***P < 0.001; **P < 0.01; *P < 0.05 (**d**)

To gain mechanistic insights into how MIEFs promote mitochondrial fusion, we first evaluated whether mitochondrial localization is required for the MIEF-mediated fusion-promoting function. To this end, we used the two cytosolic mutants MIEF1^Δ1-48^ and MIEF2^Δ1-49^ [26], lacking the first 48 or 49 N-terminal residues including the TM domain, respectively (see Fig. 1d–g). Both MIEF1^Δ1-48^ and MIEF2^Δ1-49^ were distributed in the cytosol in 293T cells (Additional file 1, Figure S7a, c). Exogenous expression of MIEF1^Δ1-48^ and MIEF2^Δ1-49^ induced mitochondrial elongation, resulting in a tubular cluster phenotype of mitochondria in WT 293T cells (Additional file 1, Figure S7a, c), similar to what was observed in Drp1^−/−^ cells (see Fig. 3a, lower panel), but not the highly compact cluster phenotype of mitochondria seen in WT 293T cells exogenously expressing either wild-type MIEF1 (Additional file 1, Figure S7a) or MIEF2 (Additional file 1, Figure S7c), in line with our previous observations [25, 26]. Next, we evaluated the effect of MIEF1^Δ1-48^ and MIEF2^Δ1-49^ on MIEF-mediated fusion using the PEG-induced cell fusion assay and the mito-PAGFP-based mitochondrial fusion assay. Compared to the empty vector control, we found that MIEF1^Δ1-48^ (Fig. 6a–d) and MIEF2^Δ1-49^ (Fig. 7a–d) did not significantly affect the mitochondrial fusion rates. These results suggest that mitochondrial localization of MIEFs is required for their mitochondrial fusion-promoting role.

Subsequently, we evaluated whether self-association properties of MIEFs were required for their fusion-promoting ability. As determined by disuccinimidyl suberate (DSS)-mediated in vivo crosslinking followed by Western blotting analysis, we confirmed that WT MIEFs can form dimers/oligomers (Additional file 1, Figure S7b, d), in line with our previous studies [25, 26]. In addition, we showed that the two deletion mutants MIEF1^Δ109-154^ and MIEF2^Δ50-104^ completely lost the ability of dimerization and oligomerization (Additional file 1, Figure S7b, d). Associated with this loss of protein self-association properties, exogenous expression of MIEF1^Δ109-154^ and MIEF2^Δ50-104^, similar to the cytosolic mutants MIEF1^Δ1-48^ and MIEF2^Δ1-49^, caused extensive mitochondrial elongation, resulting in the tubular cluster phenotype of mitochondria, but could not induce the highly compact cluster phenotype of mitochondria characteristic for overexpressed WT MIEFs (Additional file 1, Figure S7a, c). Likewise, in comparison with empty vector control, the MIEF1^Δ109-154^ (Fig. 6a–d) and MIEF2^Δ50-104^ mutants (Fig. 7a–d) did not significantly affect mitochondrial fusion neither in the PEG-induced fusion assay nor in the mito-PAGFP-based fusion assay, despite that the two mutants still retained the ability to robustly bind Drp1 as shown in Fig. 1e, g. Taken together, these data indicate that both mitochondrial localization and protein self-association, but not Drp1 binding, are required for the mitochondrial fusion-promoting ability of MIEFs. Finally, we examined whether the interaction of MIEFs with Mfn1/2 could affect the GTPase activity of Mfn1 and Mfn2 using an in vitro GTPase activity assay as previously reported [41]. To test this, Mfn1-Myc or Mfn2-Myc was immunopurified by anti-Myc agarose beads from 293T cells co-transfected with empty vector, MIEF1-V5, or MIEF2-V5 (Additional file 1, Figure S8a) and used for measurement of GTPase activity of Mfn1 and Mfn2. We found that the presence or absence of MIEF1 or MIEF2 did not significantly affect the GTPase activity of Mfn1 and Mfn2 (Additional file 1, Figure S8b).

### Elevated levels of MIEFs prevent the interaction of hFis1 with Mfn1/2 and alleviate hFis1-induced mitochondrial fragmentation

**Fig. 8:**
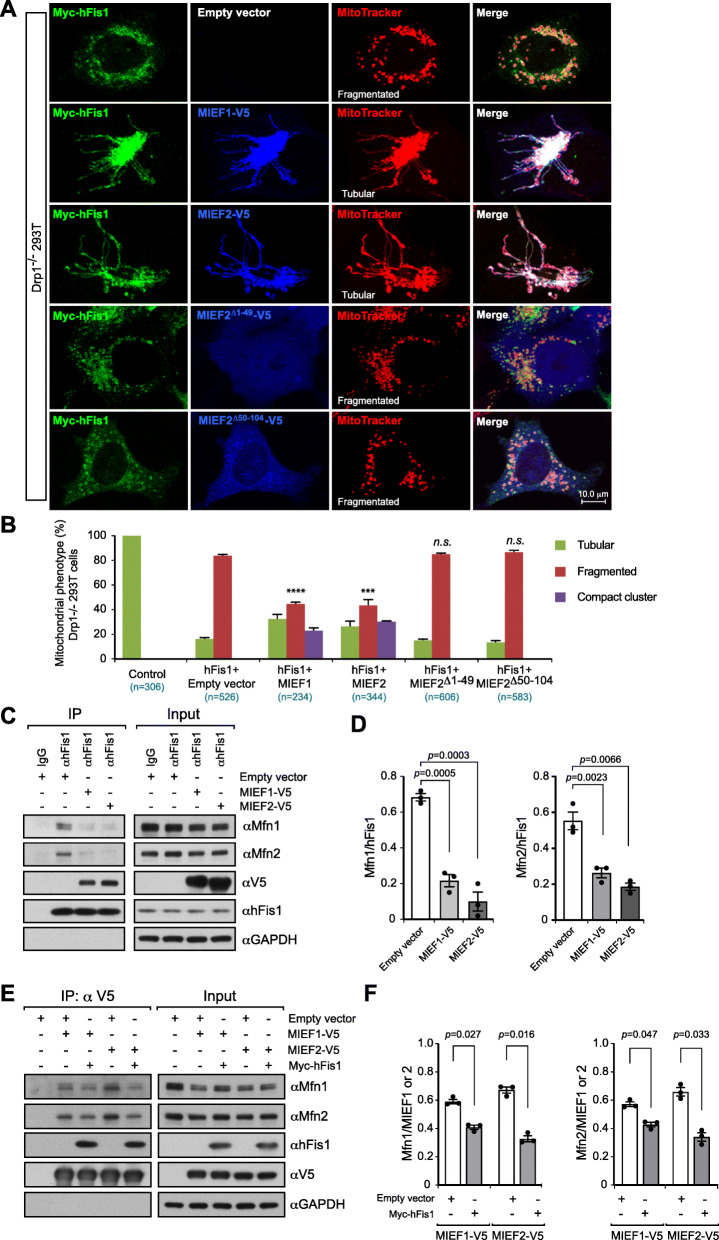
Elevated expression of MIEFs reverses hFis1-induced mitochondrial fragmentation through preventing hFis1 binding to pro-fusion proteins. **a** Elevated levels of MIEF1 or MIEF2 reverse hFis1-induced mitochondrial fragmentation in 293T cells lacking Drp1. Confocal images of mitochondrial morphologies in Drp1^−/−^ 293T cells co-transfected with Myc-hFis1 and either empty vector, MIEF1-V5, MIEF2-V5, MIEF2^Δ1-49^, or MIEF2^Δ50-104^ as indicated. The cells were stained with MitoTracker (red) followed by immunostaining with anti-Myc (green) and anti-V5 (blue) antibodies. **b** Percentages (mean ± SEM) of cells with indicated mitochondrial phenotypes in Drp1^−/−^ 293T cells co-transfected with indicated plasmids in **a**, in three independent experiments for each condition (*n* represents the total number of cells analyzed for each condition). ****P < 0.0001; ***P < 0.0005; ns, no significant. **c** Exogenous expression of MIEFs drastically decreased the interaction between hFis1 and pro-fusion proteins Mfn1 and Mfn2. 293T cells were transfected with empty vector, MIEF1-V5, or MIEF2-V5. Cell lysates were subjected to co-IP with protein G beads pre-bound to rabbit normal IgG (negative control) or anti-hFis1 antibody, followed by immunoblotting with indicated antibodies. **d** The ratios (mean ± SEM) between Mfn1 or Mfn2 and hFis1 in co-immunoprecipitates in **c** were analyzed by densitometry. **e** Elevated expression of hFis1 also impaired the interaction between pro-fusion proteins Mfn1/2 and MIEF1/2. 293T cells were co-transfected with empty vector (as negative control) and MIEF1-V5 or MIEF2-V5, as well as co-transfected with Myc-hFis1 and either MIEF1-V5 or MIEF2-V5 as indicated. Cell lysates were used for co-IP with anti-V5 beads, followed by Western blotting with indicated antibodies. **f** The ratios (mean ± SEM) between Mfn1 or Mfn2 and MIEF1-V5 or MIEF2-V5 in co-immunoprecipitates in **e** were analyzed by densitometry

It has been reported that MIEFs interact with hFis1 and Drp1 in a mutually exclusive manner, and in keeping with this, increased levels of hFis1 diminish the association of MIEFs with Drp1 via the formation of MIEF-hFis1 complexes, partially reversing MIEF1/2-induced mitochondrial elongation [25, 26]. In contrast, elevated expression of MIEF1 or MIEF2 also reduces hFis1-induced fragmentation in wild-type 293T cells [25, 26]. To analyze this competitive interaction between MIEFs and hFis1 further, we used the Drp1^−/−^ 293T cells to evaluate how MIEFs influence hFis1-induced mitochondrial fragmentation. Confocal microscopy showed that increased expression of hFis1 triggered extensive mitochondrial fragmentation even in the absence of endogenous Drp1 (Fig. 8a, upper panel; summarized in Fig. 8b), in line with our previous report that hFis1-induced mitochondrial fragmentation is Drp1 independent [31]. However, elevated expression of MIEF1 or MIEF2 significantly alleviated mitochondrial fragmentation caused by hFis1 overexpression in Drp1^−/−^ 293T cells, leading to mitochondrial elongation (Fig. 8a, middle panels; summarized in Fig. 8b), similar to what was originally observed in wild-type 293T cells co-expressing hFis1 and MIEF1 or MIEF2 [26]. In contrast, expression of either the cytosolic mutant MIEF2^Δ1-49^ or the hFis1 binding-deficient mutant MIEF2^Δ50-104^ (see Fig. 1g) led to a complete loss of the ability to reverse hFis1-induced mitochondrial fragmentation (Fig. 8a, middle and lower panels; summarized in Fig. 8b). These observations lead us to suggest that elevated expression of MIEFs can relieve the inhibitory effect of hFis1 on the fusion machinery, enhancing mitochondrial fusion irrespective of the presence or absence of Drp1. Consistent with this notion, the MIEF2^Δ50-104^ mutant did not enhance mitochondrial fusion (see Fig. 7).

**Fig. 9:**
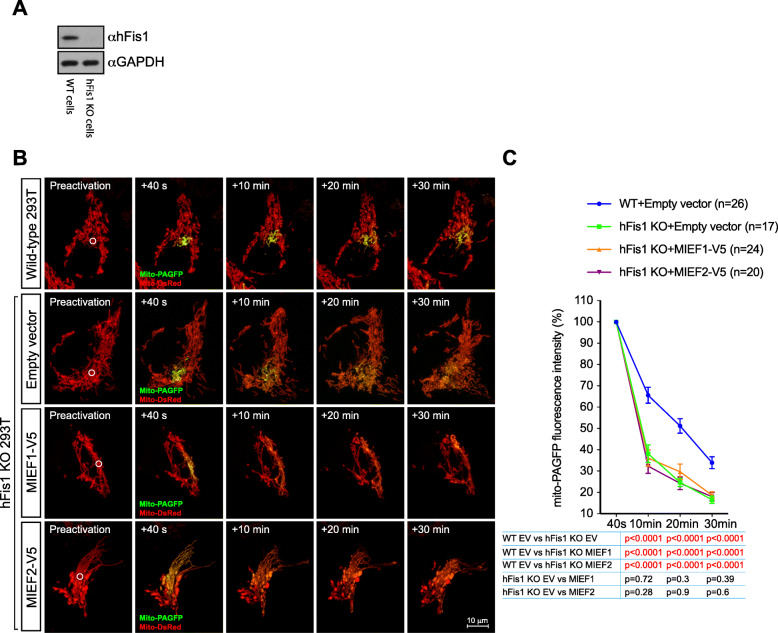
hFis1 is required for MIEF-induced mitochondrial fusion. **a** Generation of hFis1 KO 293T cell lines by CRISPR/Cas9-based genome editing was confirmed by Western blot analysis obtained from the single cell colonies derived hFis1 KO 293T cells. **b**, **c** WT 293T cells co-transfected with mito-PAGFP (0.5 μg) and mito-DsRed (0.2 μg), or hFis1 KO 293T cells co-transfected with mito-PAGFP (0.5 μg), mito-DsRed (0.2 μg), and either empty vector (0.5 μg), MIEF1 (0.5 μg), or MIEF2 (0.5 μg) were photoactivated in the ROI (white circle, 3 μm diameter) in preactivation images of mitochondria (red). After photoactivation, mito-PAGFP fluorescence (green) intensity and mitochondrial marker (mito-DsRed) were collected at indicated time points (**b**). Mitochondrial fusion was quantified by analyzing changes in fluorescence intensity of photoactivated mito-PAGFP in ROIs at 40 s and 10, 20, and 30 min. The dilution rates (percentage) of the GFP fluorescence intensity at different time points were normalized by the fluorescence intensity at 40 s after photoactivation (**c**) (*n* represents the number of cells analyzed)

We next asked whether elevated levels of MIEFs could alleviate the hFis1-induced mitochondrial fragmentation through the formation of a MIEF-hFis1 complex that would reduce the interaction of hFis1 with the pro-fusion GTPases Mfn1/2. To test this, we transiently transfected WT 293T cells with empty vector (control) or with expression plasmids encoding either MIEF1-V5 or MIEF2-V5 and evaluated the effects on the interaction of hFis1 with Mfn1/2 using co-immunoprecipitation followed by Western blotting. As shown in Fig. 8c, d, co-IP experiments revealed that elevated levels of MIEF1 or MIEF2 resulted in a significant decrease in the interaction of endogenous hFis1 with Mfn1 and Mfn2, accompanied by the interaction between hFis1 and MIEF1 or MIEF2. Similarly, elevated levels of hFis1 reduced the interaction of MIEF1 and MIEF2 with Mfn1 and Mfn2 (Fig. 8e, f). These data indicate that MIEFs and hFis1 compete for the interaction with these fusion proteins, and we conclude that increased levels of MIEFs reduce the association of hFis1 with the fusion machinery through the formation of a robust MIEF-hFis1 complex, reducing the inhibitory effect of hFis1 on the fusion machinery. To further explore this issue, we assessed MIEFs-mediated mitochondrial fusion in hFis1-deficient (hFis1 KO) 293T cells generated by CRISPR/Cas9-mediated genome editing (Fig. 9a). The mito-PAGFP-based fusion assay revealed that ablation of hFis1 enhanced mitochondrial fusion, consistent with our previous report [31]. However, exogenous expression of MIEF1 or MIEF2 in hFis1 KO cells did not seem to further enhance mitochondrial fusion and was comparable to empty vector control in hFis1 KO cells (Fig. 9b, c). This result is in line with the notion that MIEFs facilitate mitochondrial fusion via the interaction with hFis1 and reduce the inhibitory effect of hFis1 on the fusion machinery. Taken together, the data provide a novel molecular mechanism for how MIEFs orchestrate mammalian mitochondrial fusion.

## Discussion

Mitochondria are highly dynamic organelles that frequently change their morphology through continuous cycles of fission and fusion events [1, 3]. Mitochondrial elongation can be caused by activated mitochondrial fusion or/and abrogated mitochondrial fission, while the converse is true for mitochondrial fission. How the balance between fusion and fission is tuned remains only partially understood. MIEFs were originally identified as receptors for the recruitment of Drp1 to mitochondria, implicating a role in mitochondrial fission, but high expression of MIEF1/2 led to mitochondrial elongation rather than fission [25, 26], suggesting that MIEFs play a more complex role in mitochondrial dynamics. It has been proposed that MIEFs may play a dual role, regulating both fusion and fission, i.e., increased MIEF1/2 expression inhibits fission and promotes fusion, resulting in mitochondrial elongation [25], whereas other studies have argued that mitochondrial elongation following high MIEF1/2 expression is simply due to sequestration of Drp1 at mitochondria, blocking fission [27, 30].

To address this issue, we here explored the role of MIEFs in mitochondrial fusion and provide several lines of evidence that MIEFs participate in regulating the fusion machinery. Firstly, apart from the well-known interactions with pro-fission proteins Drp1, hFis1, and Mff [25, 26, 29], MIEFs also interact with the key pro-fusion GTPases Mfn1 and Mfn2, suggesting that MIEF function as a hub that balances fission and fusion (Fig. 1). Secondly, increased expression of MIEFs partially reverses Mfn1 or Mfn2 knockdown-induced mitochondrial fragmentation, but does not rescue mitochondrial fragmentation following simultaneous depletion of Mfn1 and Mfn2 or following depletion of OPA1, indicating that MIEFs promote mitochondrial fusion in an Mfn1/2- and OPA1-dependent manner (Fig. 2). Thirdly, while knockout of Drp1 inhibits the fission process resulting in a super-elongated mitochondrial phenotype, loss of Drp1 does not affect the extent of ongoing mitochondrial fusion (Fig. 3b–e). Finally, regardless of whether Drp1 is present or not, elevated levels of MIEFs efficiently facilitate mitochondrial fusion (Fig. 3f, g and Fig. 4a–d). In further support of this notion, the Drp1 binding-deficient mutants MIEF1^Δ160-169^ and MIEF2^Δ151-160^ still retain the ability to facilitate mitochondrial fusion, underscoring that the fusion-promoting role of MIEFs is independent of their role in Drp1-mediated fission (Fig. 5). Taken together, these findings suggest that MIEFs play a role not only in the fission machinery but also in the fusion machinery and exert a dual regulation of fission and fusion via interaction with both the pro-fission and pro-fusion proteins. Such a dual role of MIEFs provides a novel mechanism that may mechanistically couple the inhibition of Drp1-mediated mitochondrial division with the stimulation of Mfn1/2-mediated fusion to generate rapid changes in mitochondrial shape in response to dynamic alterations of the cellular microenvironment and mitochondrial stress.

Another important finding presented in this study is that MIEFs may participate in the tethering of adjacent mitochondria (an early step of mitochondrial fusion), most likely through MIEFs self-assembly capacity and by coordinating with mitofusins during the fusion process [12–14]. We demonstrate that mitochondrial localization and self-association are crucial for the fusion-promoting function of MIEFs. Notably, the cytosolic mutants MIEF1^Δ1-48^ and MIEF2^Δ1-49^ lost the ability to promote mitochondrial fusion. Similarly, the self-association-deficient mutants MIEF1^Δ109-154^ and MIEF2^Δ50-104^ also lost the fusion-promoting ability, albeit they still retained the ability to interact with and inhibit Drp1 function (Figs. 6 and 7). In support of a role for self-association of MIEFs in fusion, increased expression of full-length MIEFs can trigger mitochondrial aggregation resulting in the highly compact cluster phenotype of mitochondria, indicating an extensive inter-mitochondrial tethering (Fig. S7a, c). In contrast, expression of the cytosolic mutants MIEF1^Δ1-48^ and MIEF2^Δ1-49^ or the self-association-deficient mutants MIEF1^Δ109-154^ and MIEF2^Δ50-104^ (Fig. S7a, c) does not induce a highly compact cluster phenotype, in keeping with our previous observations [25, 26]. In summary, our data suggest that self-association of MIEFs may provide an additional mechanism contributing to the tethering between adjacent mitochondria in cells with high levels of MIEF1 or 2, ultimately promoting mitochondrial fusion in coordination with the action of pro-fusion GTPases Mfn1/2.

We here suggest that the interactions of hFis1 with Mfn1/2 and MIEFs have bearings on how the fusion process is regulated. hFis1 was initially identified as a pro-fission factor involved in the recruitment of Drp1 to mitochondria [42, 43], but was recently shown to robustly interact with Mfn1/2 [31]. Elevated expression of hFis1 inhibits, while knockdown of hFis1 enhances mitochondrial fusion, showing that hFis1 can trigger mitochondrial fragmentation through interaction with the pro-fusion proteins Mfn1/2 and inhibition of their GTPase activity [31]. Here, we extend this hypothesis by demonstrating that MIEFs can alleviate the inhibitory effect of hFis1 on the fusion machinery through the interaction with hFis1, thereby “shielding off” hFis1 from the association with the pro-fusion GTPases Mfn1 and Mfn2 and reducing the inhibitory role of hFis1 for the fusion machinery. Consistent with this, increased expression of MIEF1 or MIEF2 greatly reduces hFis1-induced mitochondrial fragmentation both in wild-type 293T cells [25, 26] and in Drp1^-/-^ 293T cells (Fig. 8a, b in this work). In contrast, increased expression of hFis1 also reduces MIEF-induced mitochondrial elongation [25, 26] and decreases binding of MIEF1 and MIEF2 to the pro-fusion proteins Mfn1 and Mfn2 (Fig. 8e, f). In line with these results, ablation of endogenous hFis1 facilitated mitochondrial fusion, while elevated expression of MIEF1 or MIEF2 in hFis1-deficient 293T cells did not significantly enhance mitochondrial fusion (Fig. 9). Together, these findings prompt us to suggest that MIEFs and hFis1 compete for their association with the fusion machinery, eliminating each other from the fusion machinery probably via the formation of a MIEFs-hFis1 complex. When the levels of MIEFs are high, they bind and sequester hFis1, preventing hFis1’s interaction with Mfn1 and Mfn2, permitting mitochondrial fusion.

## Conclusions

In summary, our data show that MIEFs are crucial for controlling the balance between mitochondrial fusion and fission, thereby orchestrating mammalian mitochondrial dynamics by directly engaging with both the fusion and fission machineries. In the fission machinery, elevated levels of MIEFs promote the recruitment of cytosolic Drp1 to the surface of mitochondria, where MIEFs sequester Drp1 in an inactive form, inhibiting Drp1-mediated fission leading to unopposed fusion [25, 27–29, 44]. In the fusion machinery, MIEFs interact with Mfn1/2 and facilitate mitochondrial fusion, most likely via promoting tethering of two adjacent mitochondria by self-association in coordination with Mfn1/2-mediated tethering of two outer membranes, ultimately driving mitochondrial fusion via Mfn1/2 activity. Additionally, we also show that elevated levels of MIEFs reduce the interaction of hFis1 with the pro-fusion proteins Mfn1 and Mfn2, alleviating the inhibitory effect of hFis1 on the fusion machinery.

## Methods

### Cell cultures and transfection

The HEK 293T (293T) cells and knockout 293T cells generated through the CRISPR/Cas9 system were cultured in Dulbecco’s modified Eagle’s medium (Sigma-Aldrich) with 10% (v/v) fetal bovine serum (FBS, Gibco) and 1% Penicillin-Streptomycin antibiotics (Gibco) at 37°C and 5% CO_2_.

Lipofectamine^TM^ 2000 transfection reagent (Invitrogen) was used for transient transfection of plasmids according to the manufacturer’s protocol. Expression plasmids used in this study were: MIEF1-V5, MIEF2-V5, and MIEF1/2 deletion mutants tagged by V5/His at the C-terminus including MIEF1^Δ1-48^, MIEF1^Δ50-108^, MIEF1^Δ109-154^, MIEF1^Δ173-216^, MIEF1^Δ219-289^, MIEF1^Δ296-337^, MIEF1^Δ341-463^, MIEF1^Δ345-380^, MIEF1^Δ385-425^, MIEF1^Δ431-463^, MIEF1^Δ380-463^, MIEF2^Δ1-49^, MIEF2^Δ122-132^, MIEF2^Δ151-160^, MIEF2^Δ235-250^, MIEF2^Δ265-275^ and MIEF2^Δ425-454^ [25, 26, 29], Myc-hFis1[45], and pcDNA3.1-V5/His empty vector. MIEF2^Δ50-104^ was generated by PCR and cloning into pcDNA3.1-V5 (Invitrogen). To avoid the side effect of overexpression, cells were transiently transfected with only 0.3–0.5 μg of expression plasmids for 15–17 h and harvested for further analysis.

### RNA interference (RNAi) for gene silencing

Lipofectamine^TM^ RNAiMax (Invitrogen) was used for siRNA transfection following the manufacturer’s protocol. Twenty-four hours after the first siRNA transfection, cells were re-transfected with siRNA for another 48 h and then harvested for further investigations. For siRNA treatment and transfection with plasmids, cultured cells were initially treated with siRNA twice for 48 h as described above, then transfected with expression plasmids for an additional 24 h, and finally harvested for further investigations. All siRNA oligonucleotides were purchased from Thermo Fisher Scientific and based upon the sequences as follows: human Mfn1 siRNA (Mfn1-1, 5´-GCUGG AUAGCUGGAUUGAUAAGUUU-3´), human Mfn2 siRNA (Mfn2-1 5´-GGACAAAGUUC UGCCCUCUGGGAUU-3´), human OPA1 siRNA (OPA1-1, 5´-CAGCAAUGGGAUGCAG CUAUUUAUU-3´), and Stealth RNAi™ siRNA Negative Control Kit with similar GC content as control.

### Western blotting and antibodies

Cell lysates prepared with NuPAGE^TM^ LDS sample buffer (4X) (Invitrogen) were electrophoresed on NuPAGE 4–12% Bis-Tris Gel (Life Technologies), and proteins were electrotransferred to polyvinylidene difluoride (PVDF) membranes with Trans-Blot Turbo PVDF Transfer kit (Bio-Rad). PVDF membranes were blocked with 10% skim milk (Sigma-Aldrich) in TBS, followed by incubation with indicated primary and secondary antibodies (GE healthcare). The specific proteins were detected with the Pierce ECL Western Blotting Substrate (Thermo Scientific). ImageJ was used for quantifications of bands on Western blots.

In this study, the following mouse monoclonal primary antibodies (mAbs) were used: V5-tag (#46-0705) (Invitrogen); OPA1 (#612607), Drp1 (#611113), and Myc-tag (#551101) (BD Biosciences); GAPDH (#sc-32233) (Santa Cruz); and Mitofusin 1 (#ab57602) (Abcam). Rabbit polyclonal antibodies (pAbs) were MIEF2 (#HPA042334), Fis1 (#HPA017430) (Atlas Antibodies); MIEF1 [25]; Mitofusin 2 (#9482S), PARP (#9542) (Cell Signaling); and VDAC1 (#ab15895) (Abcam). The following secondary antibodies were used: The peroxidase-conjugated anti-mouse and anti-rabbit IgG antibodies (GE Healthcare) for immunoblotting and the DyLight 488- and 649-conjugated anti-mouse and anti-rabbit IgG antibodies (Vector Laboratories) for immunofluorescence.

### Co-immunoprecipitation (co-IP)

Co-IP experiments were carried out as described [25, 46]. For chemical cross-linking followed with co-IP, cultured cells were in vivo cross-linked by 1% formaldehyde (FA) in PBS for 10 min at room temperature, and this reaction was quenched by 100 mM glycine. Thereafter, cells were suspended and sonicated in lysis buffer with 1% NP-40 and protease inhibitor cocktail complete EDTA-free (Roche Diagnostics). For co-IP without chemical crosslinking, cells were washed with PBS buffer and incubated in lysis buffer with 1% NP-40 and protease inhibitor cocktail complete EDTA-free (Roche Diagnostics) on ice for 30 min.

The cell lysates were subjected to co-IP experiments after centrifugation. For co-IP of V5-tagged exogenous MIEFs, anti-V5 agarose beads (Novus Biologicals) were incubated with the cell lysates for 2 h at room temperature. For co-IP of endogenous proteins, Dynabeads^TM^ protein G beads (Invitrogen) pre-incubated with 2 μg of antibody against target proteins were incubated with cell lysates overnight at 4°C. Next, the beads were washed with lysis buffer three times followed by PBS. The bead-conjugated proteins were dissolved in sample buffer and subjected to Western blotting.

### In vivo cross-linking with DSS

In vivo chemical cross-linking with disuccinimidyl suberate (DSS) (Thermo Fisher Scientific) was carried out as described [25, 47] with some modifications. Cells transfected with expression plasmids for 17 h were washed three times in PBS with Ca^2+^/Mg^2+^ and then incubated with 1 mM DSS or DMSO (as control) in PBS for 1 h at room temperature. The reaction was stopped with a final concentration of 20 mM Tris (pH 7.5) for 15 min at room temperature. The samples were dissolved in NuPAGE^TM^ LDS sample buffer (4X) (Invitrogen) and analyzed by Western blotting.

### Immunofluorescence (IF) and confocal microscopy imaging

Cells cultured on coverslips were washed with PBS and fixed with 4% paraformaldehyde (PFA) (Sigma-Aldrich) at room temperature for 10 min. Thereafter, cells were permeabilized with 0.5% Triton X-100, washed with PBS twice, and blocked in 2% bovine serum albumin (BSA) in PBS, subsequently incubated with indicated primary antibodies and DyLight conjugated secondary antibodies. Finally, the coverslips were mounted with the Mounting Medium with DAPI (Vector Laboratories). MitoTracker Red CMXRos (500 nM, Thermo Fisher Scientific) was used to stain mitochondria and added to cultured cells for 15 min before fixation. Confocal images were captured using the Leica TCS SP5 confocal microscopy system. For immunofluorescence and confocal experiments, different persons performed slide preparation, microscopy observation, and data analysis. Cells expressing target proteins were randomly selected and allocated into different experimental groups, and the cell number in each group was similar.

### Polyethylene glycol (PEG) cell fusion assay

PEG was purchased from VWR international. PEG-mediated cell fusion assay was performed as previously described [25, 31, 48, 49]. Briefly, 293T cells stably expressing mitoRFP (mitochondria-targeted RFP) or mitoGFP (mitochondria-targeted GFP) [31] (1:1) were co-seeded on glass coverslips for 24 h and subjected to the PEG-mediated cell fusion assay. For exogenous expression of V5-tagged MIEF1, MIEF2, or their mutants, cells stably expressing mitoRFP or mitoGFP were co-cultured for 16 h on glass coverslips and transfected with indicated plasmids or with empty vector as the control for 18 h. Before the cell fusion experiment, cells were incubated with cycloheximide (20 μg/ml, Sigma) in DMEM without serum for 30 min at 37°C to inhibit the protein synthesis. Next, cells were treated with pre-warmed (37°C) 50% (wt/vol) PEG 1500 (Sigma) solution in DMEM without serum for 90 s, then cells were washed three times with growth medium plus cycloheximide (20 μg/ml) and incubated with this medium for 5 h. Finally, cells were fixed, permeabilized, and blocked as previously described for the immunofluorescence experiments. After immunofluorescence staining with anti-V5 tag primary antibody and DyLight 649-conjugated secondary antibody, polykaryons (containing only two nuclei) were randomly selected and analyzed via the SP5 confocal microscopy system (Leica). The extent of mitochondrial fusion in hybrid cells was directly analyzed for quantitative colocalization using the Pearson’s correlation coefficient (PCC) software in the Leica TCS SP5 confocal microscopy system integrated program.

### Mito-PAGFP-based mitochondrial fusion assay

The mito-PAGFP-based assay was carried out as previously described [31, 39, 40]. For overexpression of MIEFs and their mutants, WT, Drp1^−/−^, and hFis1^−/−^ 293T cells were co-transfected with mito-PAGFP (0.5 μg), mito-DsRed (0.2 μg), and either empty vector (0.5 μg) or indicated plasmid (0.5 μg). Images were captured on a Leica TCS SP5 confocal microscope using a 63× oil objective from 16 h post-transfection. Transfected cells were recognized through mito-DsRed expression. A 3-μm-diameter circular region of interest (ROI) was marked and photoactivated by a single-pulse 405 nm laser. Images with red and green fluorescent signals were captured before and immediately following activation at 40 s and 10, 20, and 30 min. Fluorescence intensity of photoactivated mito-PAGFP in ROIs at each time point was directly analyzed by the software of the Leica Confocal System and normalized by the mito-PAGFP fluorescence at 40 s after photoactivation (shown as 100%).

### GTPase activity assay

The GTP hydrolysis activity assay was carried out as previously described [41, 50, 51]. The free r-phosphate (Pi) release of GTP was measured by the colorimetric ATPase/GTPase activity assay kit (Sigma-Aldrich). 293T cells transiently co-transfected with Mfn1-Myc or Mfn2-Myc and either empty vector, MIEF1-V5, or MIEF2-V5 for 20–22 h were washed twice with phosphate-free buffer (10 mM Tris-HCl pH 7.5, 150 mM NaCl) and lysed in phosphate-free lysis buffer (10 mM Tris-HCl pH 7.5, 150 mM NaCl, 1% NP-40, protease inhibitor cocktail complete EDTA-free, Roche Diagnostics) on ice for 1 h. After centrifugation, the supernatants were subjected to immunoprecipitation (IP) with 40 μl of anti-Myc tag agarose bead (Abcam) for 2h at room temperature. After washing with lysis buffer 3 times and twice with 0.5 M Tris-HCl pH 7.5, beads were re-suspended in assay buffer provided with the kit and subjected to GTPase activity assay according to the manufacturer’s protocol. The absorbance at 620 nm was read for all samples. Anti-Myc tag agarose beads incubated with the cell lysate prepared from 293T cells transfected with empty vector were used as the mock control in the GTPase activity assay. To evaluate the quantities of input immunopurified proteins for GTPase activity assay, the proteins binding with anti-Myc tag agarose beads were subjected to Western blot analysis.

### Statistical analysis

Statistical analysis of differences between two experimental groups was performed by the Student’s *t* test online software (http://www.physics.csbsju.edu/stats/t-test.html). *P* values of less than 0.05 were regarded as statistically significant.

## Supplementary Information

**Additional File 1: Figures S1-8.** **Figure S1.** MIEFs robustly interact with Mfn1 and Mfn2 in the absence of chemical crosslinking. **Figure S2.** Multiple sequence alignment of MIEF1 orthologs in different vertebrate species. **Figure S3.** Multiple sequence alignment of MIEF2 orthologs in different vertebrate species. **Figure S4.** Overexpression of MIEFs and deletion mutants does not induce apoptosis as assessed by western blot analysis with PARP antibody. **Figure S5.** Knockdown of Mfn1, Mfn2 or OPA1 by siRNA. **Figure S6.** Exogenous expression of MIEF1 in MIEF1 KO 293T or MIEF2 in MIEF2 KO 293T cells enhances mitochondrial fusion. **Figure S7.** Mitochondrial localization and dimerization/oligomerization of MIEFs are required for their fusion-promoting ability. **Figure S8.** MIEFs do not affect the GTPase activities of Mfn1 and Mfn2.

**Additional File 2.** Source data

## Data Availability

All data generated or analyzed during this study are included in this published article and its supplementary information files.

## Abbreviations

BSA: Bovine serum albumin
Co-IP: Co-immunoprecipitation
DSS: Disuccinimidyl suberate
DR: The disordered regions
Drp1: Dynamin-related protein 1
Mff: Mitochondrial fission factor
MIEF1/2: Mitochondrial elongation factors 1 and 2
Fis1: Mitochondrial fission 1
FA: Formaldehyde
Mfn1/2: Mitofusin 1 and 2
MOM: Mitochondrial outer membrane
MIM: Mitochondrial inner membrane
mitoGFP: Mitochondria-targeted GFP
mitoRFP: Mitochondria-targeted RFP
mito-PAGFP: Mitochondrial matrix-targeted photoactivatable green fluorescent protein
PFA: Paraformaldehyde
PCC: Pearson’s correlation coefficient
PEG: Polyethylene glycol
PVDF: Polyvinylidene difluoride
RNAi: RNA interference
TM: Transmembrane
VDAC1: Voltage-dependent anion channel 1
WT: Wild type
