# Supplementary material for: MIEF1/2 orchestrate mitochondrial dynamics through direct engagement with both the fission and fusion machineries
Source: BMC Biol. 2021 Oct 21;19:229. doi: 10.1186/s12915-021-01161-7 (PMC8532385; doi:10.1186/s12915-021-01161-7)
Supplement: Supplementary file 1 — Additional File 1: Figures S1-8. Figure S1. MIEFs robustly interact with Mfn1 and Mfn2 in the absence of chemical crosslinking. Figure S2. Multiple sequence alignment of MIEF1 orthologs in different vertebrate species. Figure S3. Multiple sequence alignment of MIEF2 orthologs in different vertebrate species. Figure S4. Overexpression of MIEFs and deletion mutants does not induce apoptosis as assessed by western blot analysis with PARP antibody. Figure S5. Knockdown of Mfn1, Mfn2 or OPA1 by siRNA. Figure S6. Exogenous expression of MIEF1 in MIEF1 KO 293T or MIEF2 in MIEF2 KO 293T cells enhances mitochondrial fusion. Figure S7. Mitochondrial localization and dimerization/oligomerization of MIEFs are required for their fusion-promoting ability. Figure S8. MIEFs do not affect the GTPase activities of Mfn1 and Mfn2. [file 12915_2021_1161_MOESM1_ESM.pdf]

Supplemental information

MIEF1/2 orchestrate mitochondrial dynamics through direct engagement with both the fission and fusion machineries

Rong Yu[1], Tong Liu[1], Shao-Bo Jin[2], Maria Ankarcrona[3], Urban Lendahl[2], Monica Nistér[1*†], and Jian Zhao[1*†]

Supporting information

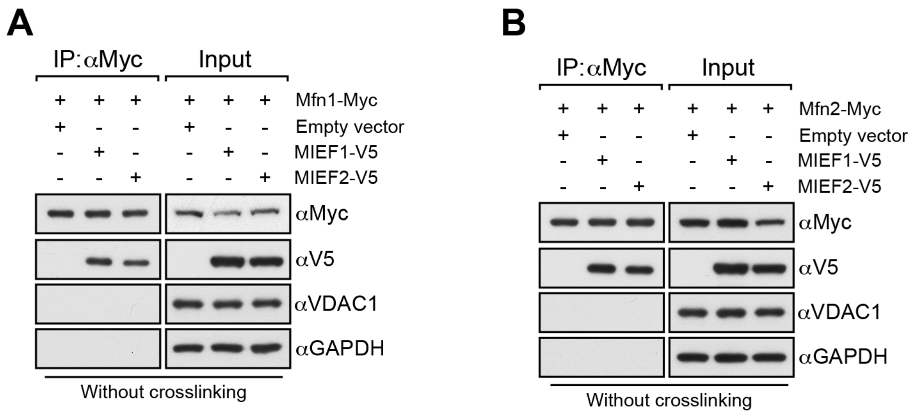

Figure S1. MIEFs robustly interact with Mfn1 and Mfn2 in the absence of chemical crosslinking. a 293T cells were co-transfected with Mfn1-Myc and either empty vector (as control), MIEF1-V5 or MIEF2-V5. Cell lysates were used for co-IP with anti-Myc beads, followed by Western blotting with indicated antibodies. **b** 293T cells were co-transfected with Mfn2-Myc and either empty vector (as control), MIEF1-V5 or MIEF2-V5. Cell lysates were used for co-IP with anti-Myc beads, followed by Western blotting with indicated antibodies.

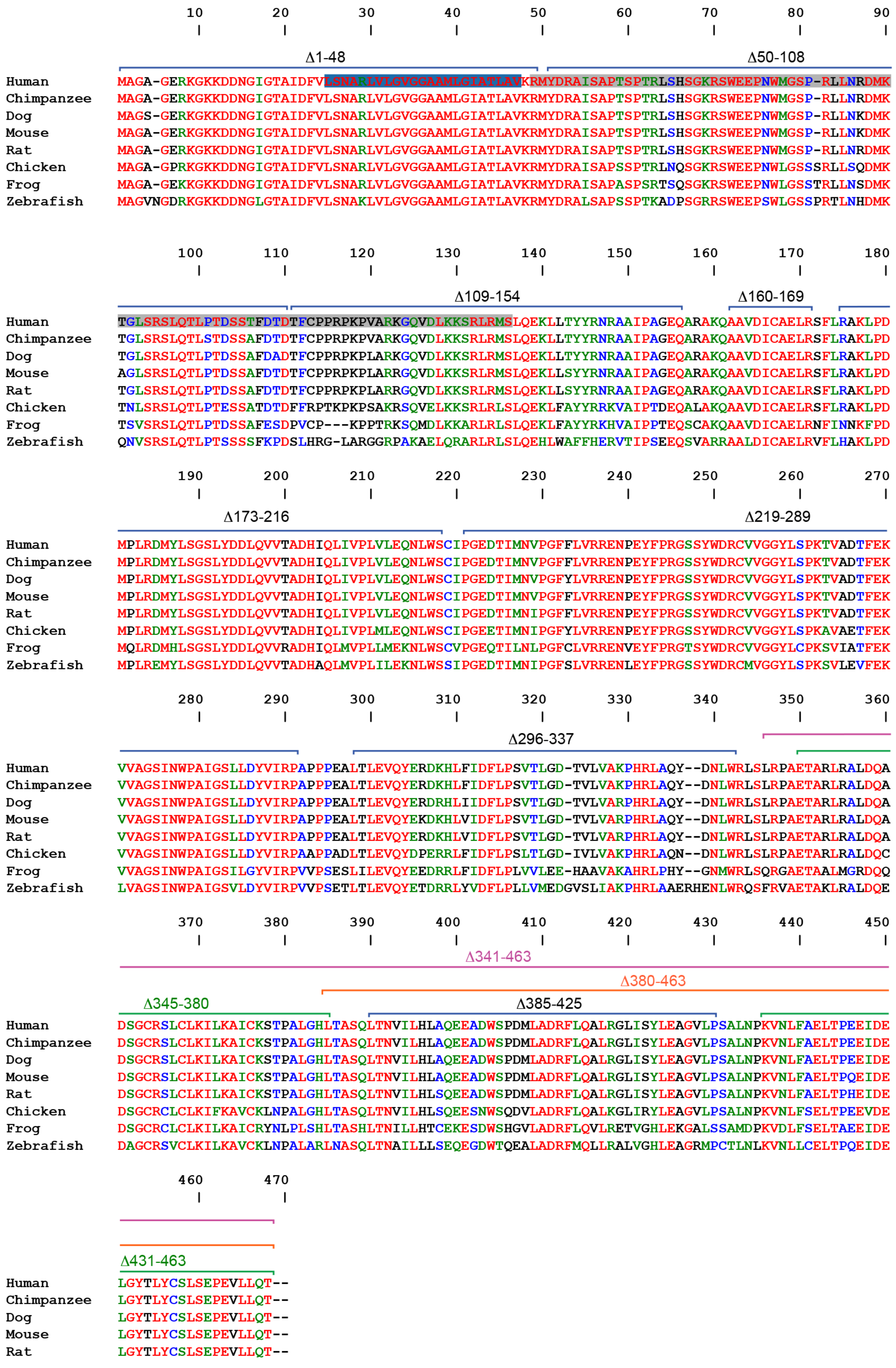

Figure S2. Multiple sequence alignment of MIEF1 orthologs in different vertebrate species. The amino acid sequence alignment was generated by the CLUSTALW program (https://npsa-prabi.ibcp.fr). The predicted transmembrane domain (blue color) and the disordered region (grey color) are indicated in human MIEF1. The GenBank numbers are:

Human (NP_061881), Chimpanzee (XP_001165282), Dog (XP_852489), Mouse (NP_848834), Rat (NP_001007710), Chicken (XP_416248), Frog (NP_001025698), Zebrafish (NP_001077309). Red: identical; Green: strongly similar; Blue: weakly similar; Black: different amino acids. The different MIEF1 mutants used in this work are indicated above the alignment.

```
                   10        20        30        40        50        60        70        80        90
                    |         |         |         |         |         |         |         |         |
                              Δ1-49                                                    Δ50-104
Human      MAEFSQKRGKRRSDEGLGSMVDFLLANARLVLGVGGAAVLGIATLAVKRFIDRATSPRDEDDTKAD------SWKELSLLKATPHLQPRP
Orangutan  MAEFSQKRGKRRGDEGLGSMVDFLLANARLVLGVGGAAVLGIATLAVKRFIDRATSPRDEDDTKAD------SWKELSLLKATPHLQPRP
Mouse      MAEFSQKQRKQSGSEGLGSVVDFLLANARLVLGVGGAAVLGIATLAVKRLIDRATSPPDEDDTKGD------SWKELSLLRATSPQKPQP
Rat        MAEFSQKQRKQHGGEGLGSVVDFLLANARLVLGVGGAAVLGIATLAVKRLIDRATNPRDEDDTKGD------SWKELSLLRATSHPKPQP
Hamster    MAEFSRKQRKQRGSEGLGSVVDFLLANARLVLGVGGAAVLGIATLAVKRLIDRATSPRDEDDAKGD------SWKELSLLRATSHQQPQP
Ursus      MAEFSQKRGKQRD-EVLGGTVDFLLANARLVLGVGGAAVLGIATLAVKRLIDRATSPRDEDDAKGDNTCLEESWKELSLLKATPRQQPRT
Walrus     MAEFSQKRGKQRD-EVLSGTMDFLLANARLVLGVGGAAVLGIATLAVKRLIDRATSPRDEDDAKGDSTCLEESWKELSLLKATPRQQPRT
Whale      MAEFSQNRGKRRDGEVLGGAVDFLLANARLVLGVGGAAVLGIATLAVKRLIDRATSPRDEDDAKRDSTCLEDSWQELSLLKATPRLQPQP

                  100       110       120       130       140       150       160       170       180
                    |         |         |         |         |         |         |         |         |
                                                   Δ122-132                    Δ151-160
Human      PPAALSQPVLPLAPSSSAPEGPAETDPEV--TPQLSSPAP-LCLTLQERLLAFERDRVTIPAAQVALAKQLAGDIALELQAYFRSKFPEL
Orangutan  PPAALSQPVLPLAPSSSAPEGPAKSDPEV--TPQLSSPAP-LCLTLQERLLAFERDRVTIPAAQVALAKQLAGDIALELQAYFQSKFPEL
Mouse      PPAAFSQPLATGSPSPSVPVEPTPIHSPT--TPKFSTIAP-LCLTFQERLLAFERKHVITPEAHVTLAKQLAGDIALELQAYLRSKFPEL
Rat        SPAAFSQPKSPVSPSPSAPVGPTPTHSQT--TPKLSSVAP-LCLTFQEKLLAFERNHVIVPEAHVTLVKQLAGDIGLELQAYLRSKFSEL
Hamster    PPAALSQPTPLVSPSPSAPVEPTPAHSQTTQTPQRSSLAP-LCLTFQEKLLAFERNHVIVPEARVALVKQLAGDIALELQAYLRSKFPEL
Ursus      PPAALSQPVSLAAPSPAAPAGSADAGPQM--SPPLSSPAP-LCLTFQEKLLAFERDHVTIPAAHVALAKQLAGDIALELQAYLRNKFPEL
Walrus     PPAALSQPLSLPGPSPASPEGPTDAGPQM--SPPPSSPAP-LCLTFQEKLLAFEQDHVTIPAAQVALAKQLAADIALQLQAYLRNKFPEL
Whale      RPTALSQPVLPPAPSPSAPEGPADADPPT--SPQLCSPVPSVCLTFQEKLLAFERDHVTMPVAHVALAKQLAGDIALELQAYLRNKFPEL

                  190       200       210       220       230       240       250       260       270
                    |         |         |         |         |         |         |         |         |
                                                                               Δ235-250
Human      PFGAFVPGGPLYDGLQAGAADHVRLLVPLVLEPGLWSLVPGVDTVARDPRCWAVRRTQLEFCPRGSSPWDRFLVGGYLSSRVLLELLRKA
Orangutan  PFGAFVPGGPLYDGLQAGAADHVRLLVPLVLEPGLWSLVPGVDTVARDPRCWAVRRTQLEFCPRGSSPWDRFLVGGYLSSRVLLELLRKV
Mouse      PFGALVPGGPLYDGLQAGTAEHVRLLAPLELEPGLWSLVPGVDTVAREPRCWAVRRTQLEFHPRGCSPWDRFLVGGYLSSRVLLELLRKA
Rat        PFGVLVPSGPLYEGLQAGTAEHVRLLVPLELEPGLWSLVPGMDTVAREPRCWAVRRTQLEFHPRGCSPWDRFLVGGYLSPRVLLELLRKA
Hamster    PFGALVPGGPLYDGLQAGAAERVRLLAPLELEPDLWSLVPGMDTVAREPRCWAVRRTQLEFHPRGRSPWDRFLVGGYLSSRVLLELLRKA
Ursus      PFGALVPSGPLYDGLQAGAADRVRLLVPLALEGGLWSLVPGLDTVARDPRCWAVRRTQLEFWPRGSSPWDRFLVGGYLSARLLLELLRKA
Walrus     PFGALVPSGPLYDGLQAGAADHVRLLVPLALEGGLWDLVPGLDTVARDPRCWAVRRTQLEFRPRGSSPWDRFLVGGYLSPRVPLELLRKA
Whale      PFGALMPGGPLYDGLQAGAADPVRLLVPLVLEPGLWSLVPGVDTVAQDPRCWAVRRTQLEFHPRGSSPWDRFLVGGYLSPRVLLELLRKA

                  280       290       300       310       320       330       340       350       360
                    |         |         |         |         |         |         |         |         |
              Δ265-275
Human      LAASVNWPAIGSLLGCLIRPSMASEELLLEVQHERLELTVAVLVAVPGVDADDRLLLAWPLEGLAGNLWLQDLYPVEAARLRALDDHDAG
Orangutan  LAASVNWPAIGSLLGCLIRPSMASEELLLEVQHERLELTVAVLVAVPGVDADDRLLLAWPLEGLAGNLWLQDLYPVEAARLRALDDRDAG
Mouse      LSASVNWPAIGSLLGCLIWPDVASEELLLKVQHECLEFTLAVLMVVPGASTDDRLLLAWPLEGLASNLWLQDLYPVETARLRALDDQDAG
Rat        LSASVNWPAIGSLLGCLIWPNVASDELLLKVHHECLEFTLAVLMVVPGASTDDRLLLAWPLEGLASNLWLQDLYPVETARLRALDDQDAG
Hamster    LAASVNWPAIGSLLGCLIRPNVASEELLLEVQHGSLEFTLAVLMVVPGASTDDRLLLAWPLEGLAANLWLQDLYPVEVACLRALDDQDAG
Ursus      LASSINWPAIGGLLGCLIRPSVASEELLLEVQHERLELTVAVLLAVPSAAVEEPLLLAWPLEGLAGNLWLQDWYPAEAARLRALDERDTG
Walrus     LASSVNWPAIGGLLGCLIRPSVASEEPLLEVQHECLELTVAVLLAVTGAAAEDPVLLAWPLEGLAGSLWLQDWYPAEAARLRALDVRDAG
Whale      LTASVNWPAIGSLLGCLIRPHVASEELLLEVQHECLELPVAVLLSTAGAQAGD-LLLAWPLEGLAGNLWLQDWYPAEAARLRALDERDAG

                  370       380       390       400       410       420       430       440       450
                    |         |         |         |         |         |         |         |         |
                                                                                            Δ425-454
Human      TRRRLLLLLCAVCRGCSALGQLGRGHLTQVVLRLGEDNVDWTEEALGERFLQALELLIGSLEQASLPCHFNPSVNLFSSLREEEIDDIGY
Orangutan  TRRRLLLLLCAVCRGCSALGQLGRGHLTQVVLRLGEDNVDWTEEALGERFLQALELLIGSLEQASLPCHFNPSVNLFSNLREEEIDDIGY
Mouse      TRRRLLLLLCGICRGHPALVRLGWSHLTQVVLHLGEEEVAWTEEALGERFLQALEFLVGSLEQASLPCHFNPSVNLLGNFREEEIDDIGY
Rat        TRRRLLLLLCGVCRGHPVLVRLGWSHLTQVILRLGEEEVAWTEEALGERFLQALEFLVGSLEQASLPCHFNPSVNLLGSFREEEIDDIGY
Hamster    TRRRLLLLLCGVCRGHPALVHLGWSHLTQVVLHLGKEEVAWTEEALGDRFLQALEFLVGSLEQASLPCHFNPSVNLLGSFREEEIDDIGY
Ursus.     SRRRLLRLLCGVCRGHPALGRLDRSHLSQVVLHLGEAEADWAEAALGERFLQALEALIDSLERASLPCHFNPDVNLFGGLRAEEIDDLGF
Walrus     ARRRLLRLLCGVCRGHPALARLDRCHLSQVVLRLGEAEADWAEAALGQRFLQALEALVHSLERASLPHHFNPGVNLFGGLRAEEIDALGF
Whale      TRRRLLLLLCGVCRGHPALGRLGRGPLTQVVLHLGEQEADWAEEALAEHFLQALELLVGSLERASLPCHFSRGVNLLDGLQEEDIDDMGC

                  460       470
                    |         |

Human      ALYSGLQEPEGLL-----------
Orangutan  ALYSGLQEPEGLL-----------
Mouse      VLYSGLQVPESLF-----------
Rat        VLYSGLQVPESLF-----------
Hamster    LLYSGLQVPERLLQVDEDRDWLVF
Ursus      ALYSGLQAPEWLL-----------
Walrus     ALYRGLRAPERLL-----------
Whale      ALYWGLRAPEGLL-----------
```

Figure S3. Multiple sequence alignment of MIEF2 orthologs in different vertebrate species. The amino acid sequence alignment was generated by the CLUSTALW program (https://npsa-prabi.ibcp.fr). The predicted transmembrane domain (blue color) and the disordered region (grey color) are indicated in human MIEF2. The GenBank numbers are: human (NP_631901), Orangutan (NP_001125789), Mouse (NP_001009927), Rat (XP_001077609), Hamster (A0A1U7QI09), Ursus (A0A3Q7XHE2), Walrus (A0A2U3WA35), Whale (A0A2Y9MBG5). Red: identical; Green: strongly similar; Blue: weakly similar; Black: different amino acids. The different MIEF2 mutants used in this work are indicated above the alignment.

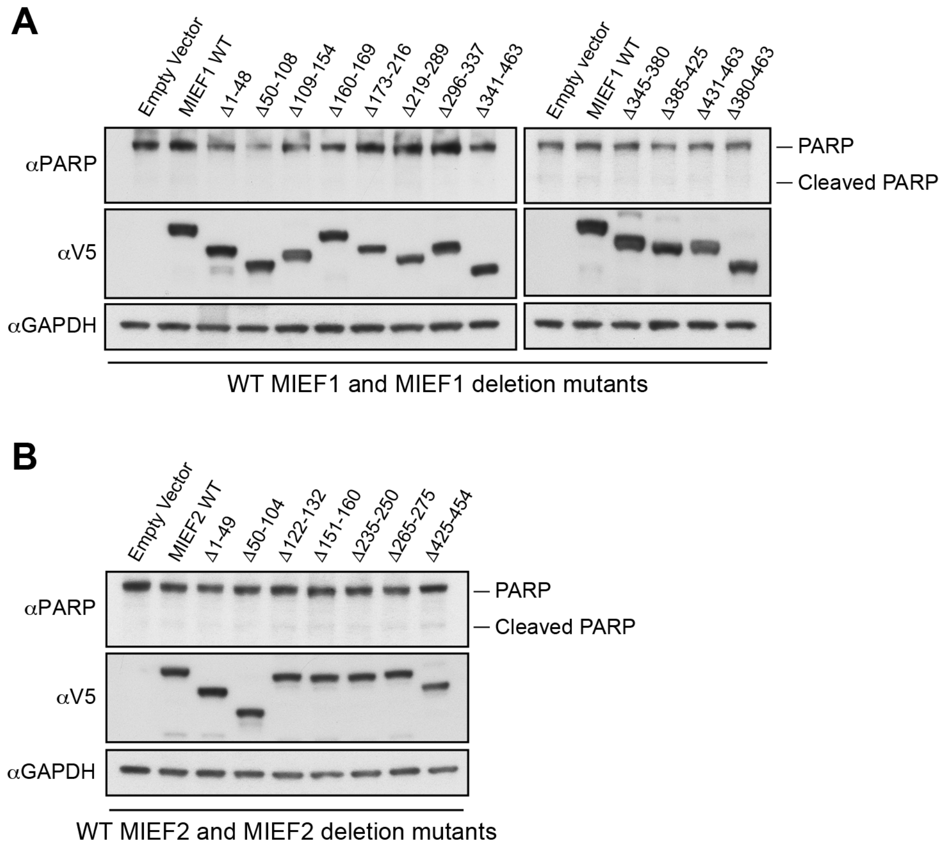

Figure S4. Overexpression of MIEFs and deletion mutants does not induce apoptosis as assessed by western blot analysis with PARP antibody. a WT 293T cells were transfected with 0.5 µg of either empty vector, MIEF1-V5 or V5-tagged deletion mutants. Cells were harvested after transfection for 20 h and analyzed by Western blotting with indicated antibodies. **b** WT 293T cells were transfected with 0.5 µg of either empty vector, MIEF2-V5 or V5-tagged deletion mutants. Cells were harvested after transfection for 20 h and analyzed by Western blotting with indicated antibodies.

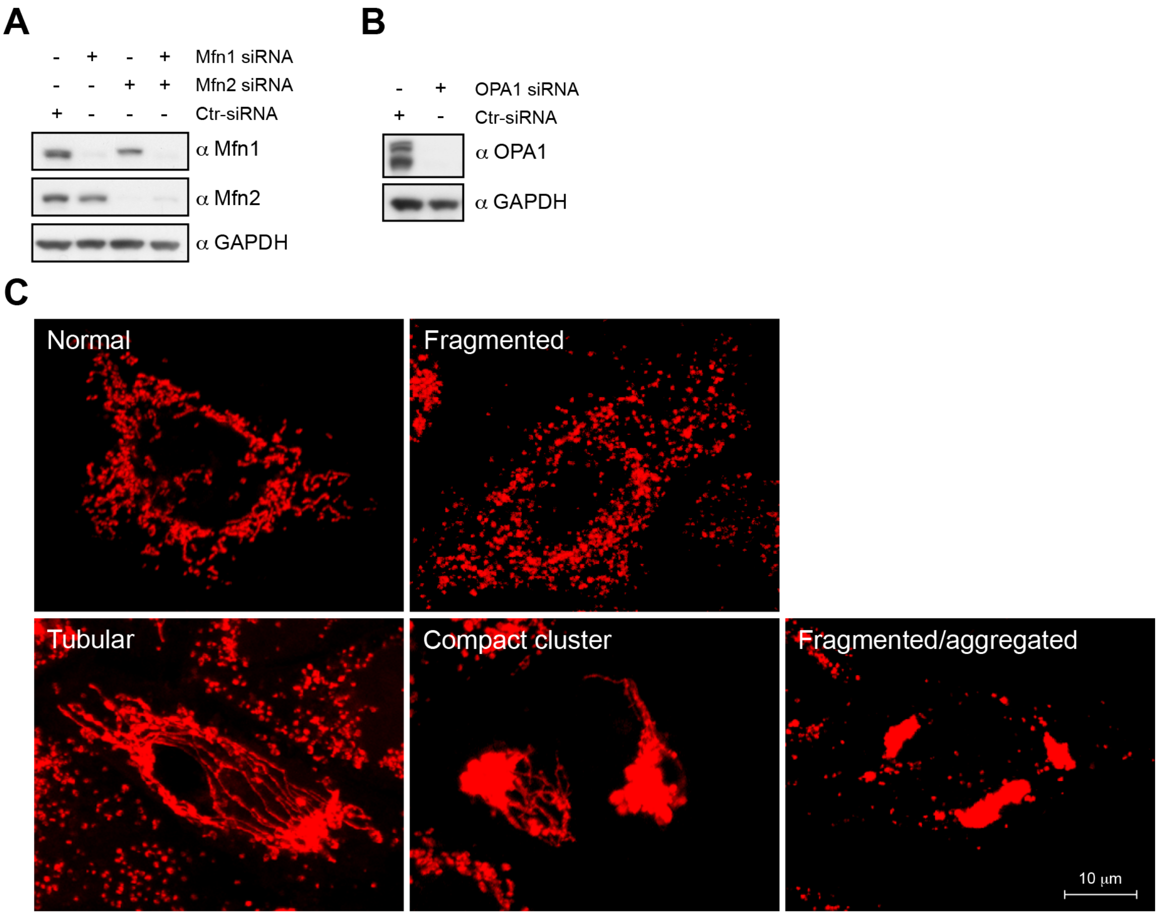

Figure S5. Knockdown of Mfn1, Mfn2 or OPA1 by siRNA. a, **b** 293T cells were treated with control siRNA (Ctr-siRNA), Mfn1-, or Mfn2-specific siRNA (**a**) and with control siRNA (Ctr-siRNA) or OPA1-specific siRNA (**b**) for 72 h, and then cell lysates were subject to immunoblotting analysis with indicated antibodies. **c** Mitochondrial phenotypes with normal (mix of tubular and round forms), fragmented, tubular, compact cluster and fragmented/aggregated forms are illustrated. Mitochondria were stained with MitoTracker Red CMXRos.

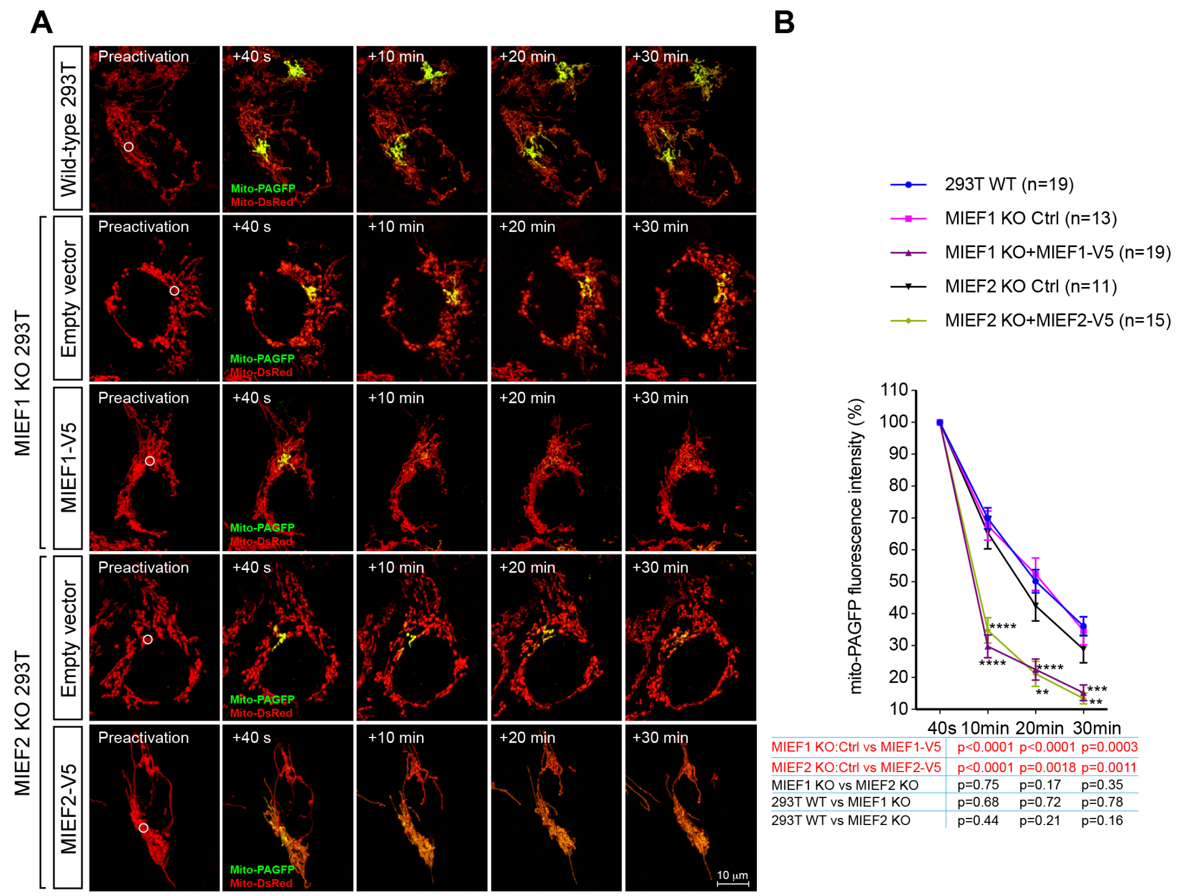

MIEF1 KO:Ctrl vs MIEF1-V5	p<0.0001	p<0.0001	p=0.0003
MIEF2 KO:Ctrl vs MIEF2-V5	p<0.0001	p=0.0018	p=0.0011
MIEF1 KO vs MIEF2 KO	p=0.75	p=0.17	p=0.35
293T WT vs MIEF1 KO	p=0.68	p=0.72	p=0.78
293T WT vs MIEF2 KO	p=0.44	p=0.21	p=0.16

Figure S6. Exogenous expression of MIEF1 in MIEF1 KO 293T or MIEF2 in MIEF2 KO 293T cells enhances mitochondrial fusion. a WT 293T cells co-transfected with mito-PAGFP (0.5 µg) and mito-DsRed (0.2 µg) (upper panel), and MIEF1 KO 293T (middle panel) or MIEF2 KO 293T cells (lower panel) co-transfected with mito-PAGFP (0.5 µg), mito-DsRed (0.2 µg), and either empty vector (0.5 µg), MIEF1 (0.5 µg) or MIEF2 (0.5 µg), were photoactivated in the ROI (white circle, 3 µm diameter) in preactivation images of mitochondria (red). After photoactivation, images of cells with mito-PAGFP fluorescence (green) and mitochondrial marker (mito-DsRed) were collected at indicated time points. **b** Mitochondrial fusion was quantified by analyzing changes in fluorescence intensity of photoactivated mito-PAGFP in ROIs at 40 sec, 10, 20 and 30 min. The dilution rates (percentage) of the GFP fluorescence intensity at different time points were normalized by the fluorescence intensity at 40 sec after photoactivation (**b**) (*n* represents the number of cells analyzed, ** $p<0.01$, *** $p<0.001$, ****$p<0.0001$).

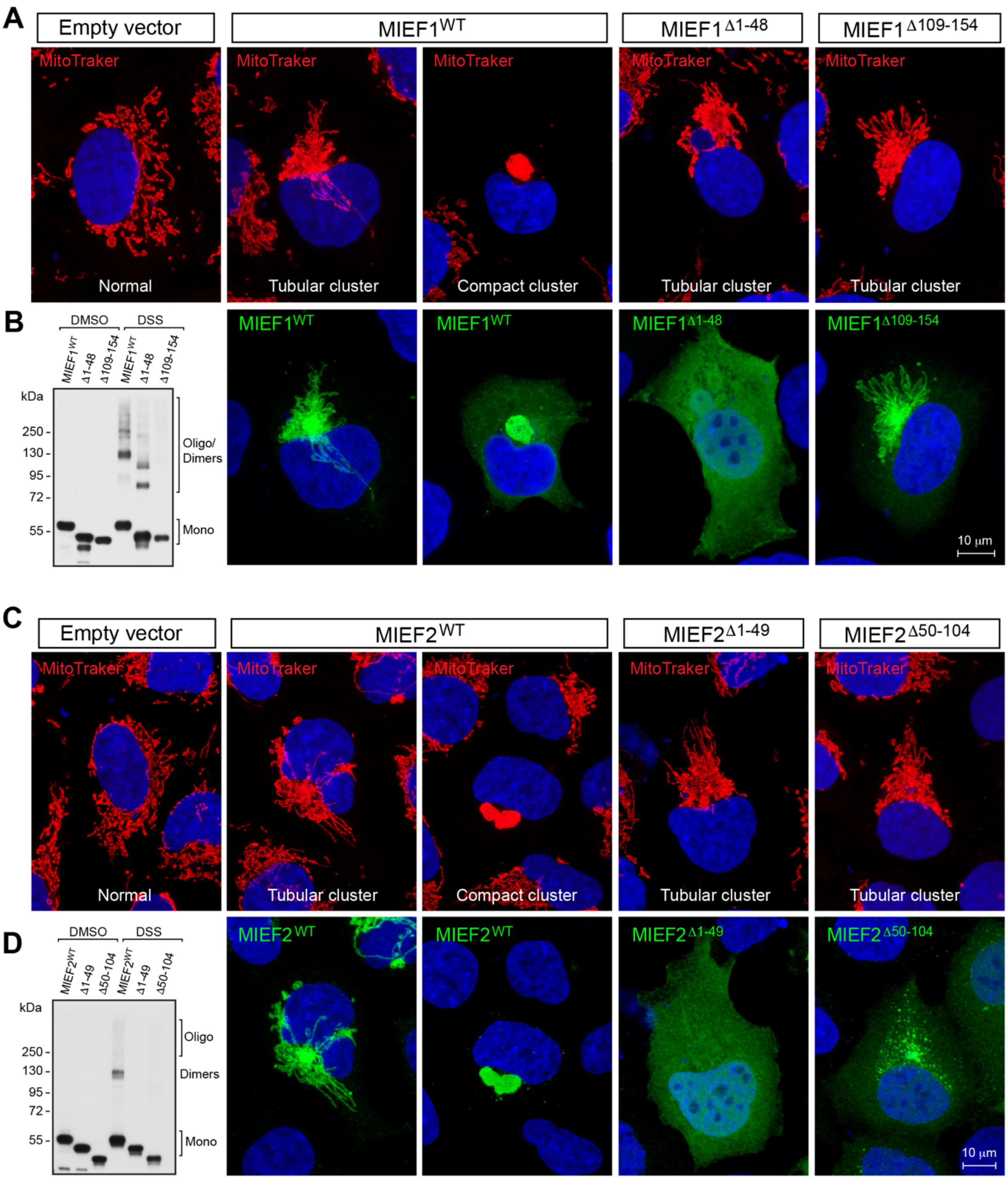

Figure S7. Mitochondrial localization and dimerization/oligomerization of MIEFs are required for their fusion-promoting ability. a Confocal image of mitochondrial morphology in 293T cells transfected with empty vector (Control), MIEF1-V5, MIEF1$^{\Delta 1\text{-}48}$-V5 or MIEF1$^{\Delta 109\text{-}154}$-V5. The cells were stained with MitoTracker (red) followed by immunostaining with anti-V5 (green) antibody. **b** 293T cells were transfected with MIEF1-V5, MIEF1$^{\Delta 1\text{-}48}$-V5 or MIEF1$^{\Delta 109\text{-}154}$-V5 and incubated with DMSO (as control) or 1 mM DSS *in vivo* chemical crosslinking for 1h at room temperature, and then the cell lysates were subject to immunoblotting analysis with anti-V5 antibody. Oligo, oligomers; Mono, monomers. **c** Confocal images of mitochondrial morphology in 293T cells transfected with empty vector (Control), MIEF2-V5, MIEF2$^{\Delta 1\text{-}49}$-V5 or MIEF2$^{\Delta 50\text{-}104}$-V5. The cells were stained with

MitoTracker (red) followed by immunostaining with anti-V5 (green) antibody. **d** 293T cells were transfected with MIEF2-V5, MIEF2$^{\Delta 1\text{-}49}$-V5 or MIEF2$^{\Delta 50\text{-}104}$-V5 and incubated with DMSO (as control) or 1 mM DSS *in vivo* chemical crosslinking for 1 h at room temperature, and then the cell lysates were subject to immunoblotting analysis with anti-V5 antibody. Oligo, oligomers; Mono, monomers.

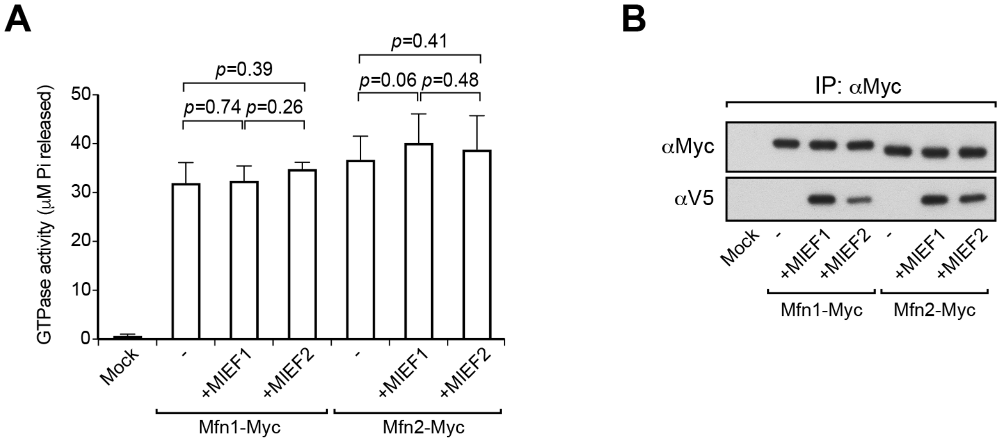

Figure S8. MIEFs do not affect the GTPase activities of Mfn1 and Mfn2. a, b GTP hydrolysis activities of Mfn1 and Mfn2 in the presence or absence of MIEF1 or MIEF2 were determined by measuring the concentration of free phosphate (P_i) released from GTP. Myc-tagged Mfn1 or Mfn2 and either empty vector, MIEF1-V5, or MIEF2-V5 were transiently expressed separately in 293T cells and immunopurified by anti-Myc agarose beads. GTPase activities of immunopurified Myc-tagged Mfn1 and Mfn2 were determined (**a**). The input levels of immunopurified Mfn1-Myc and Mfn2-Myc as well as co-immunoprecipitated MIEF1 or 2 with Mfn1 or 2 were assessed by immunoblotting with indicated antibodies (**b**).
