# Supplementary material for: MIEF1/2 orchestrate mitochondrial dynamics through direct engagement with both the fission and fusion machineries
Source: BMC Biol. 2021 Oct 21;19:229. doi: 10.1186/s12915-021-01161-7 (PMC8532385; doi:10.1186/s12915-021-01161-7)

Figure 1A Source data

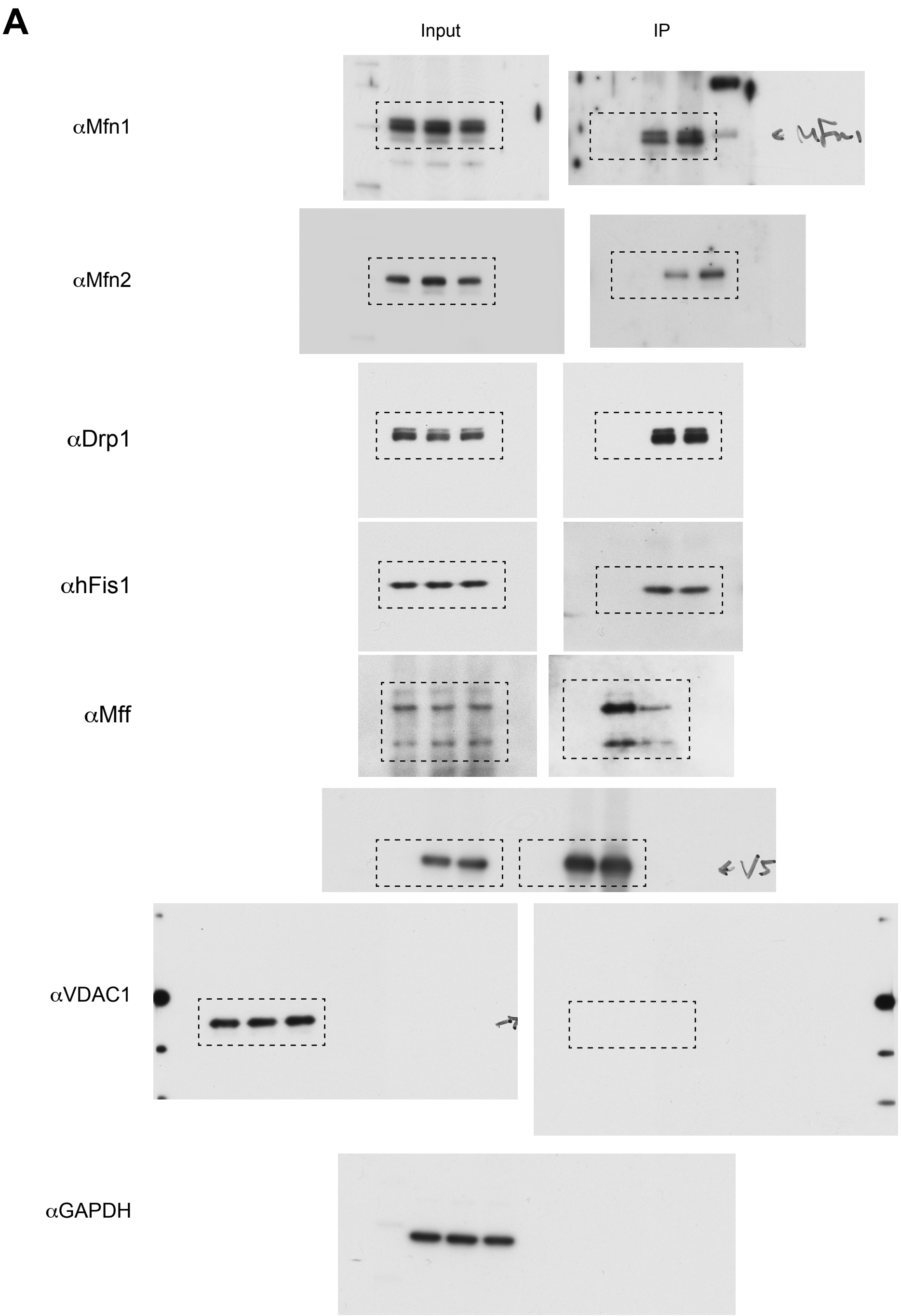

Figure 1B C Source data

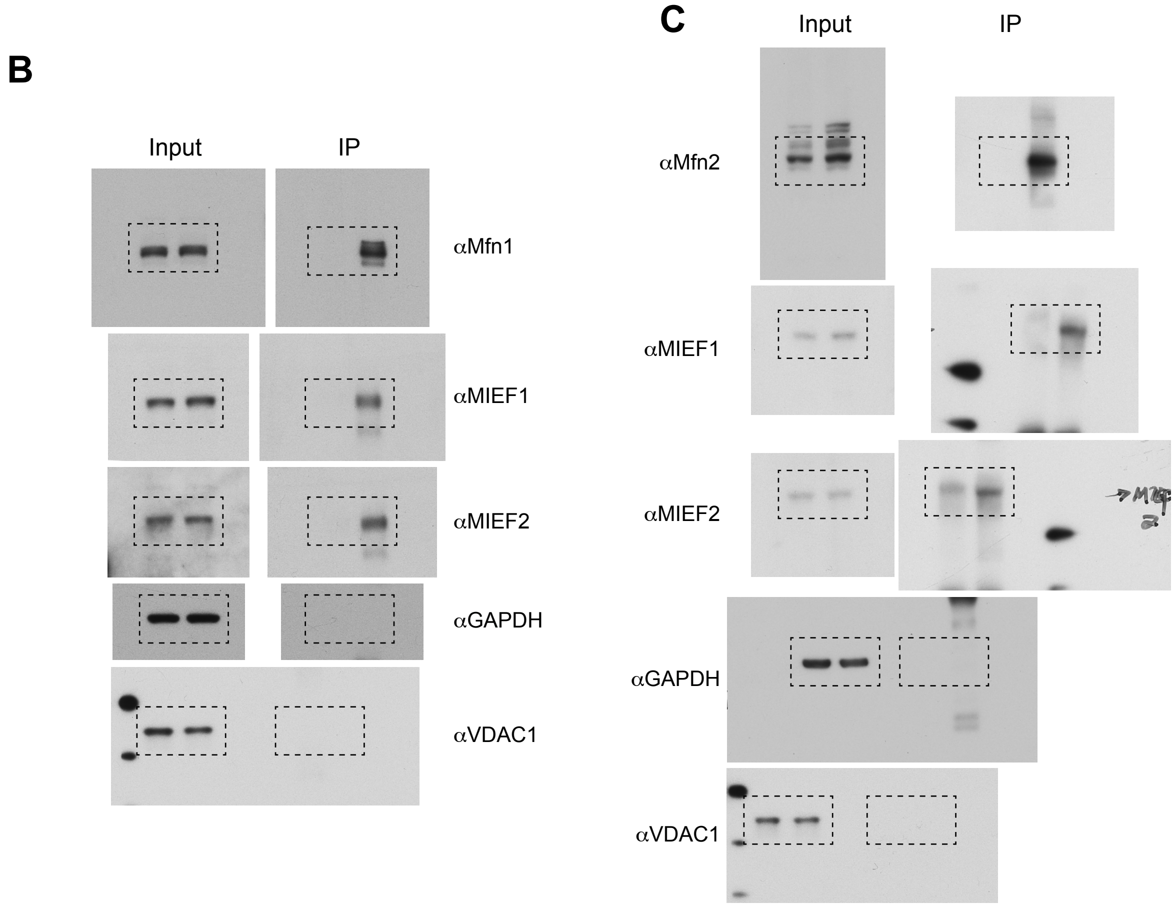

Figure 1E source data

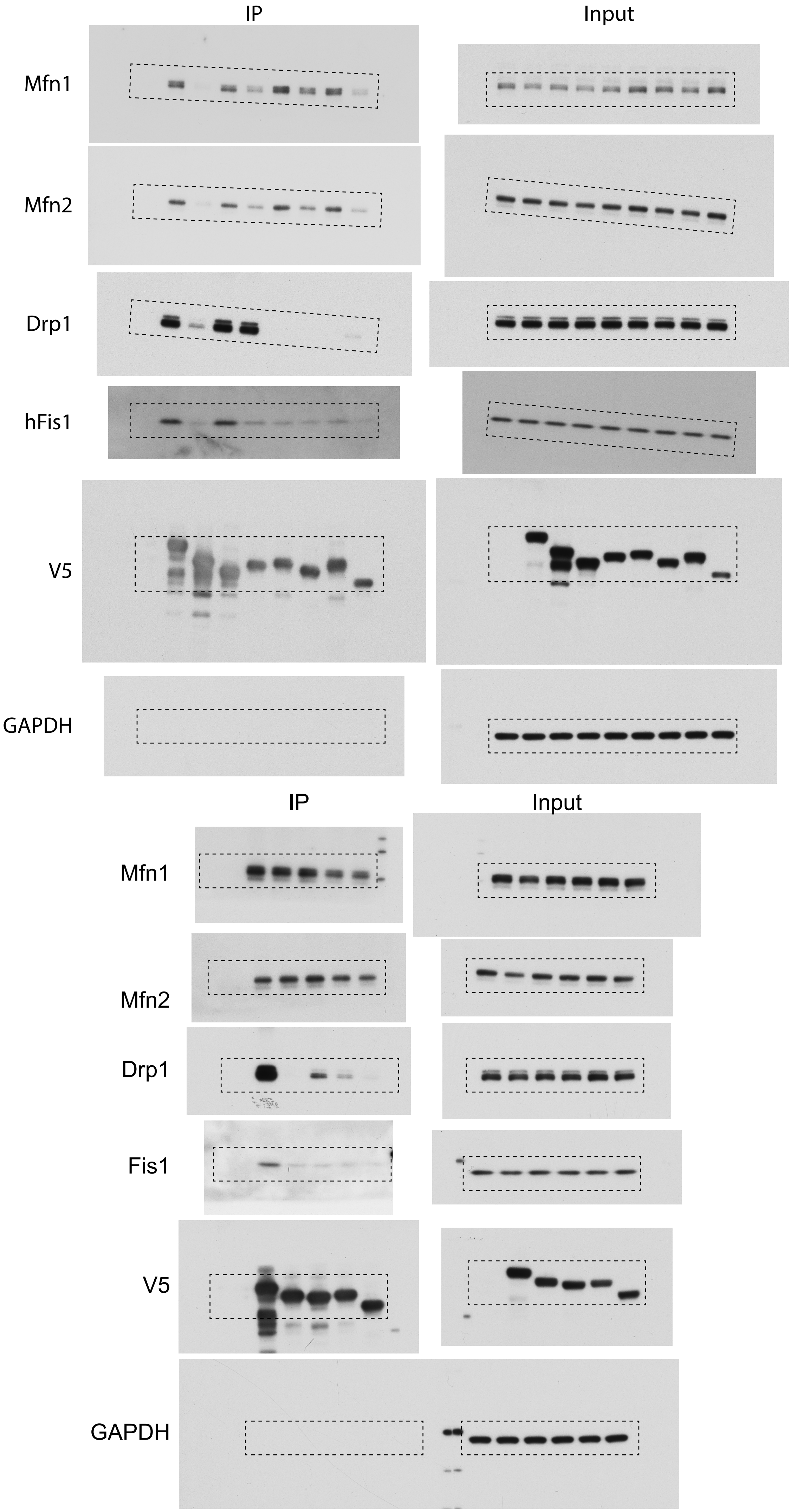

Figure 1G Source data

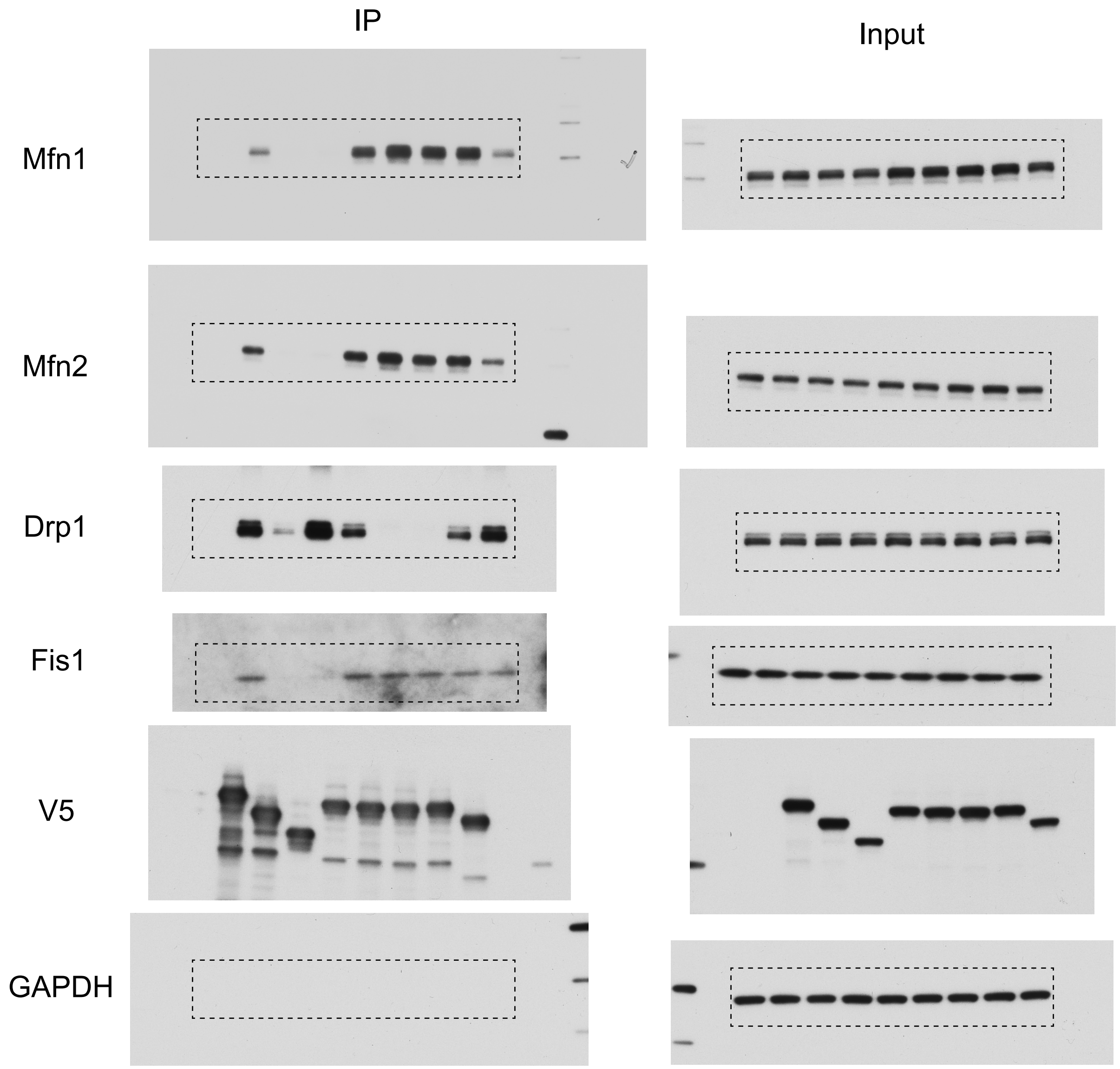

Figure 5B source data

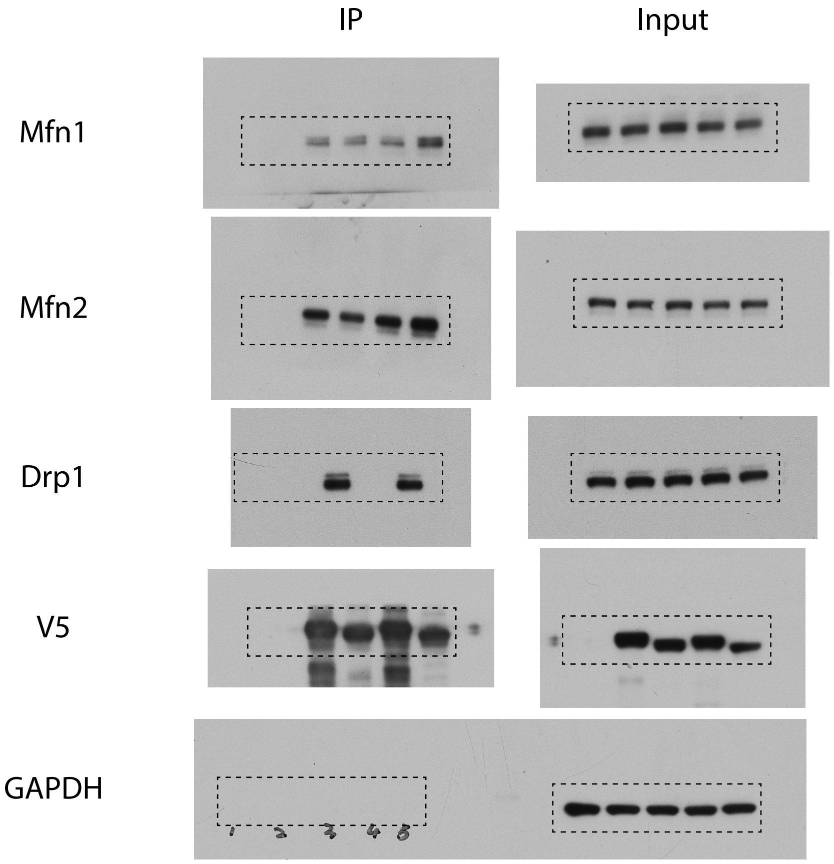

Figure 8C E Source data

C

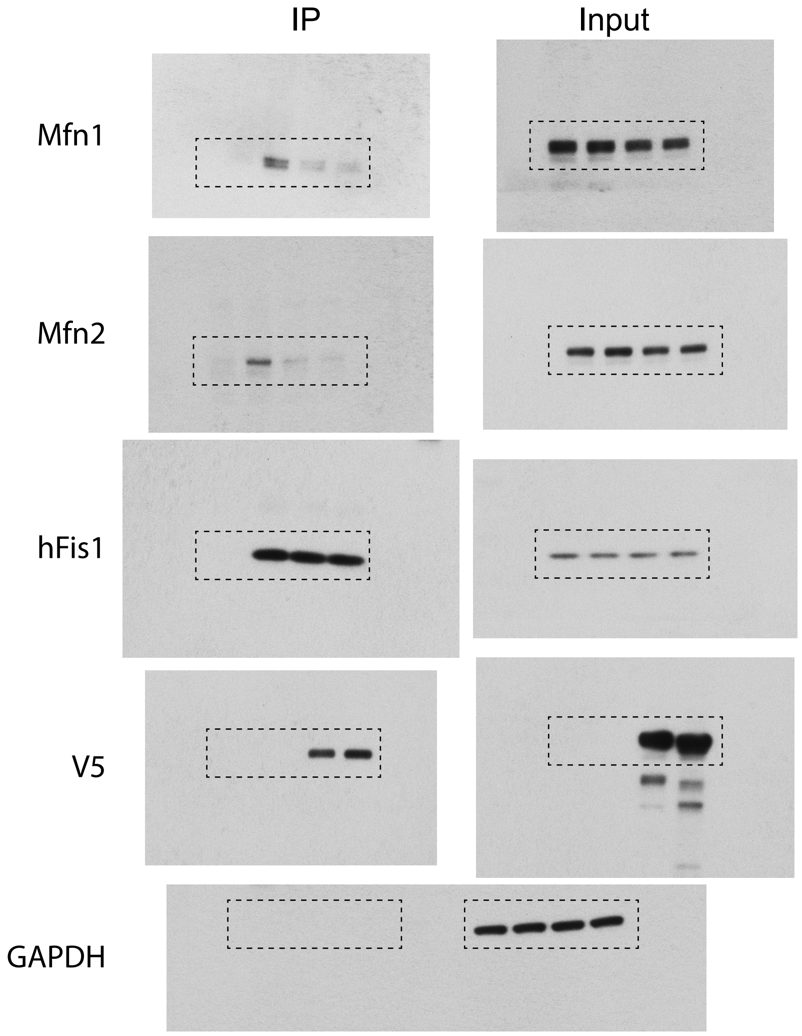

E

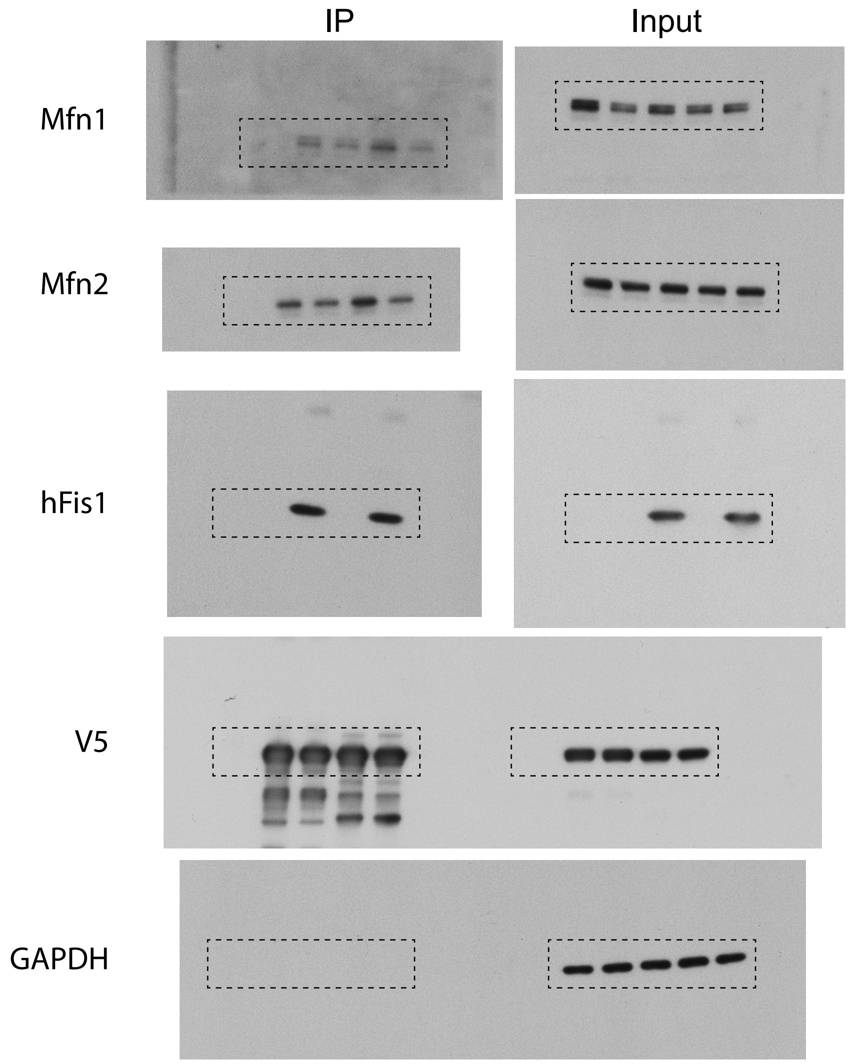

Figure 9A Source data

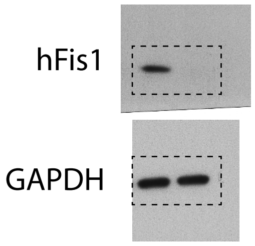

Figure S1A B Source data

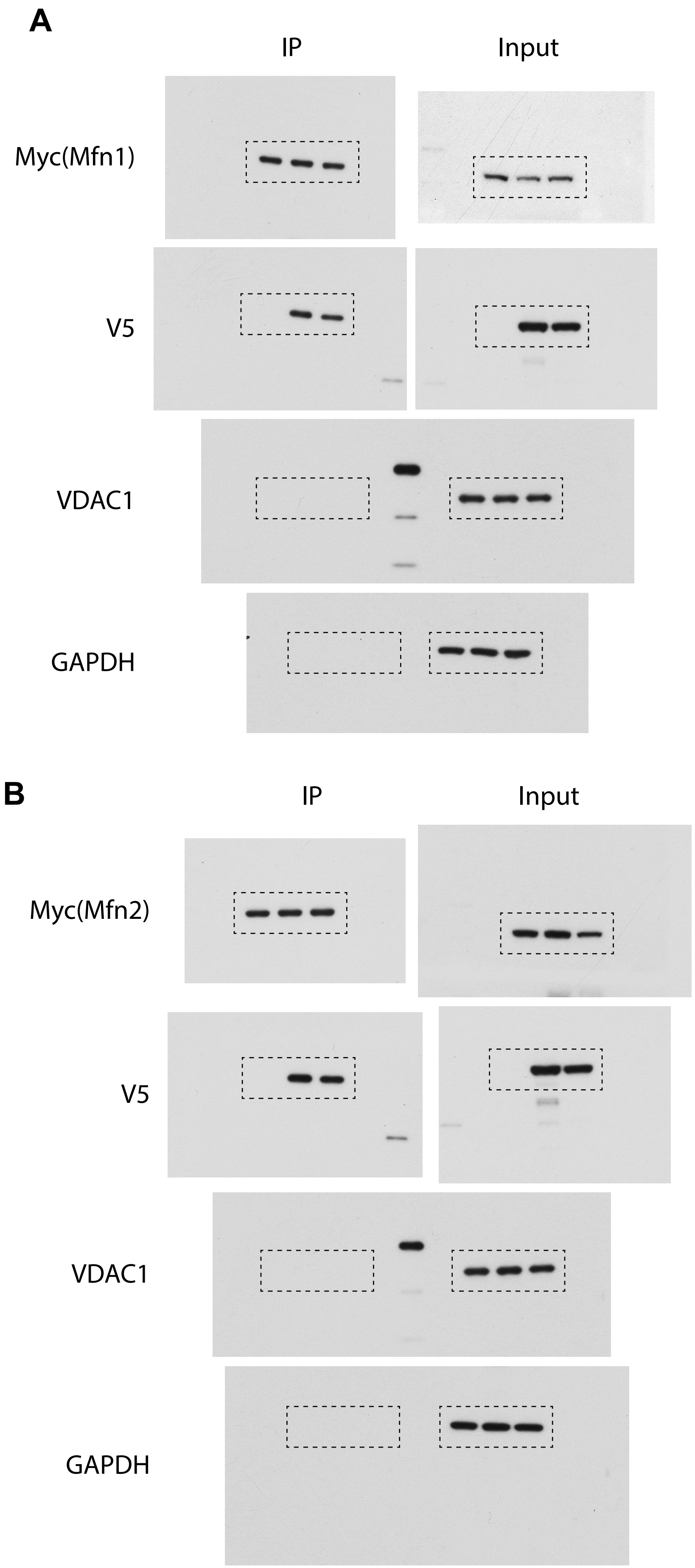

Figure S4 A B Source data

A

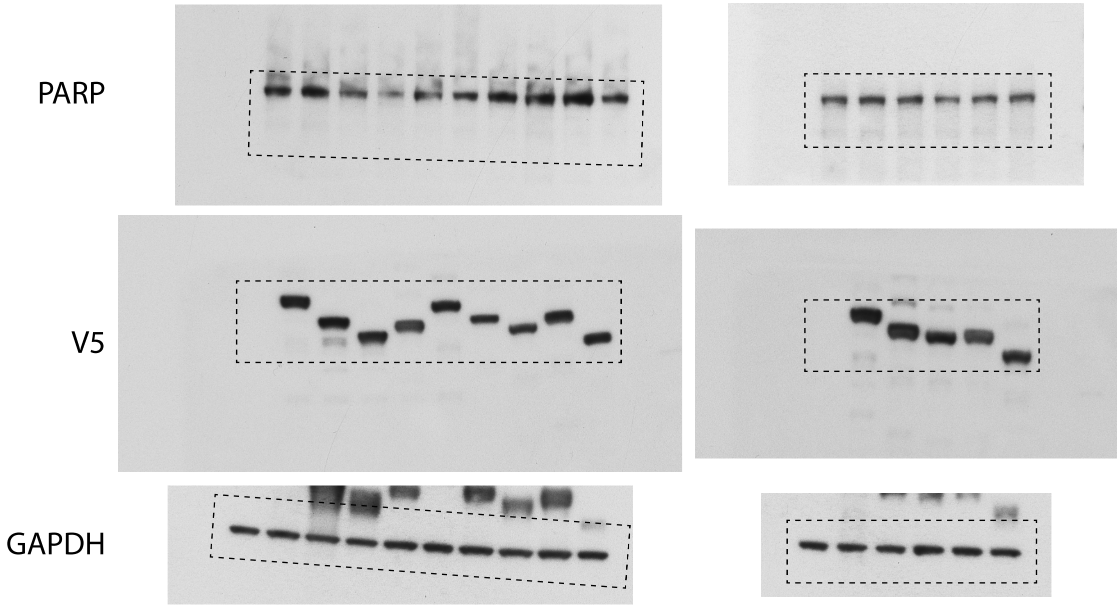

B

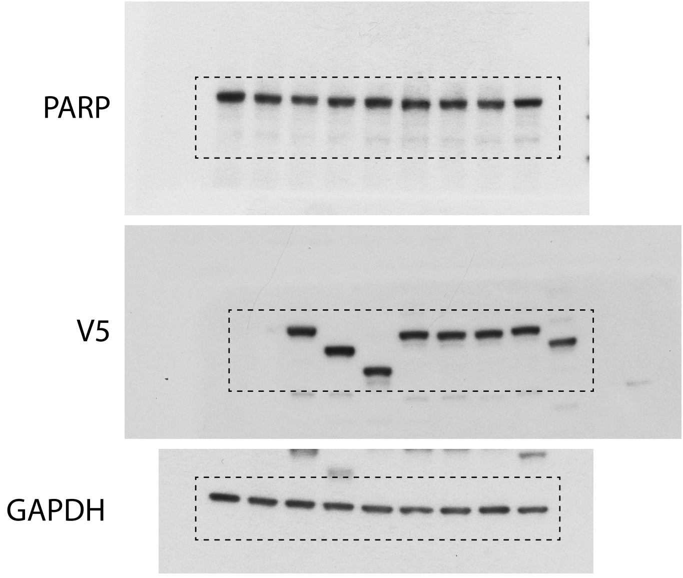

Figure S5A B Source data

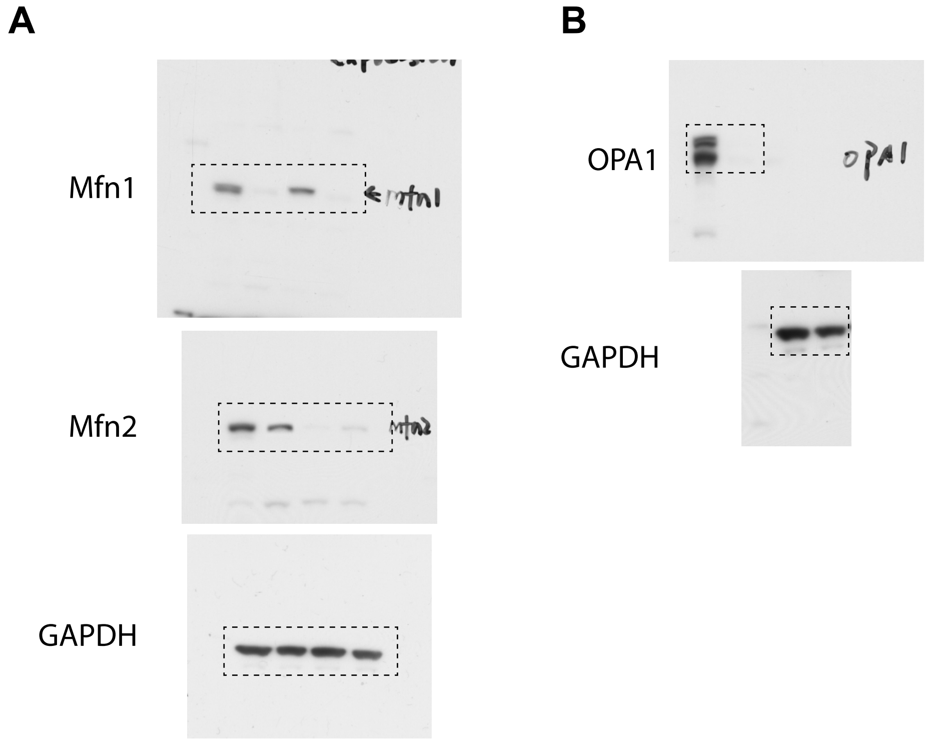

Figure S8B Source data

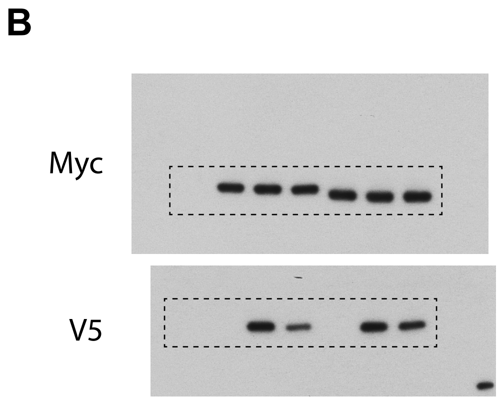

Supplement: Supplementary file 2 — Additional File 2. Source data [file 12915_2021_1161_MOESM2_ESM.pdf]
